# Overcoming serious incompatibilities resulting from intergeneric hybridisation between *Diplotaxis tenuifolia* and *Brassica oleracea* to develop CMS wall-rocket

**DOI:** 10.3389/fpls.2026.1872935

**Published:** 2026-08-07

**Authors:** Thomas Nothnagel, Barbara Sell, Otto Schrader, Johannes Wolff, Bright Enogieru Osatohanmwen, Holger Budahn

**Affiliations:** 1 Institute for Breeding Research on Horticultural Crops, Julius Kuehn-Institute (JKI), Federal Research Centre for Cultivated Plants, Quedlinburg, Germany; 2 phenoLytics GmbH, Timmendorfer Strand, Germany

**Keywords:** 3D X-ray CT, *Brassica oleracea*, cytoplasmic male sterility, *Diplotaxis tenuifolia*, flow cytometry, GISH, intergeneric hybridization, molecular markers

## Abstract

Alloplasmic male-sterile breeding lines of *Diplotaxis tenuifolia* were developed by intergeneric hybridisation between cytoplasmic male-sterile cauliflower (*Brassica oleracea* L.) and perennial wall-rocket. Amphidiploid filial generation (F_1_) plants (2*n* = 2*x* = 20, CD) were obtained by manual crosses and ovary culture. The phenotype of the hybrids was mostly similar to that of the wall-rocket parent. The plants were male-sterile, but absolutely no seed set was achieved after pollination with *D. tenuifolia*. Amphitetraploid F_1_ hybrid plants (4*n* = 4*x* = 40, CCDD) obtained by colchicine treatment were backcrossed seven times with pollen of the *Diplotaxis* parent to develop alloplasmic diploid (2*n* = 2*x* = 22, DD) *D. tenuifolia* lines. The reduction of *Brassica* chromosomes was recorded by flow cytometry and chromosome counting. Ovary culture was essential up to the Backcross generation 4 (BC_4_ generation) because of absent or disturbed seed development. From the BC_5_ generation onward, no further embryo rescue was necessary. The developed *D*. *tenuifolia* lines are completely stable with regard to their cytoplasmic male sterility (CMS) status. Fertile or partially fertile pollen was observed neither in the F_1_ generation nor in any of the subsequent backcross generations (BC_1_ to BC_7_). Male sterility was attributed to both premeiotic (sporophytic) and postmeiotic (pollen-abortive) effects, similar to those observed in the CMS *Brassica oleracea* parent. Regarding morphological traits and growth dynamics, only variations in the dimensions and dissection of leaves between different cross progenies were observed. The plant phenotype, vigour, and yield potential of the CMS lines (BC_7_) are comparable to those of the *D. tenuifolia* parental line. The female fertility has been only moderately restored; however, the proportion of normally shaped seeds per pod is still lower than that of the *Diplotaxis* control. A significant number of the developed seeds showed disturbed and shrunken embryogenic tissue, which could be visualised and measured in more detail by 3D X-ray CT. The reasons for these developmental abnormalities need to be investigated in further studies. No further incompatibilities were observed between the used CMS-inducing cybrid cytoplasm and the *D. tenuifolia* genome. Therefore, the used CMS-inducing cybrid cytoplasm has been proven to be a vital source of CMS for *D. tenuifolia*, and the developed lines are applicable for hybrid breeding programs.

## Highlights

- Alloplasmic male-sterile breeding lines of *Diplotaxis tenuifolia* were developed by intergeneric hybridisation of wall-rocket salad with CMS *Brassica oleracea*, followed by seven recurrent backcross generations and determination of their breeding value.

## Introduction

The trend of Mediterranean cuisine has promoted the market presence of leafy greens and, particularly, the production of rocket salads. From the medical point of view, species of the genera *Eruca* and *Diplotaxis* are highly interesting because of their remarkably high contents of bioactive compounds such as glucosinolates, vitamin C, and flavonoids (Martínez-Sánchez et al., 2008; D’Antuono et al., 2009; Guijarro-Real et al., 2017). These compounds are assumed to have preventive effects against cancer and cardiovascular diseases (Traka and Mithen, 2009; Lenzi et al., 2014; Petropoulos et al., 2017).

Two decades ago, *Eruca sativa* Mill. was the prevalent rocket species, but consumer preference shifted in the meantime to the perennial wall-rocket due to its smoother leaves and unique taste (Caruso et al., 2018). Hence, research and breeding activities have been intensified for *Diplotaxis tenuifolia* L. regarding the evaluation of genetic resources (Bozokalfa et al., 2011; Coelho et al., 2022), tolerance to biotic (Caruso et al., 2018; Coelho et al., 2022) and abiotic stress factors (De Vos et al., 2013; Cavaiuolo et al., 2017; Petrini et al., 2023), as well as the content of pharmaceuticals (Pasini et al., 2012; Durazzo et al., 2013; Bell and Wagstaff, 2014, 2019) and their positive or negative association to taste (D’Antuono et al., 2009; Di Gioia et al., 2018; Bell et al., 2020).

The genus *Diplotaxis* belongs, together with *Brassica*, *Raphanus*, *Sinapis*, and *Eruca*, to the economically highly important Brassicaceae family. Hybrid breeding using self-incompatibility (SI) or cytoplasmic male sterility (CMS) is the major breeding method for *Brassica napus* (rapeseed), *B. oleracea* (cabbage), *Brassica rapa* (pak choi), *Brassica pekinensis* (Chinese cabbage), and *Raphanus sativus* (radish). Among at least nine available types of cytoplasmic male sterility (reviewed by Ren et al., 2022), the Ogura-CMS system is the most widely applied. Originally, it was identified in a Japanese landrace of *Raphanus sativus* (Ogura, 1968). Alloplasmic *Brassica* lines were developed by interspecific crosses between this donor and *B. napus* and *B. oleracea*, followed by a multigeneration backcross programme (Bannerot et al., 1974). These plants showed male sterility but also undesirable traits such as disturbed nectary development and a chlorotic leaf habit when grown at temperatures lower than 15°C (Bannerot et al., 1977). A replacement of the *Raphanus* chloroplasts by those of *Brassica* has been performed by protoplast fusion to overcome these problems (Pelletier et al., 1983). The Ogu-INRA^®^ system of male sterility is characterised by the complete abortion of pollen development in nearly normally developed anthers (Renard et al., 1992). Histological studies revealed a premature breakdown of the tapetum cell layer, as also occurs in radish plants with Ogu cytoplasm. This results in impaired pollen development at the microspore stage in the absence of functional mitochondria (González-Melendi et al., 2008). The mitochondrial open reading frame *orf138* was confirmed to be responsible for mitochondrial malfunction (Bonhomme et al., 1991, 1992; Singh et al., 2019). In addition to rapeseed, the Ogu-CMS system is broadly used in *B. oleracea*, *B. rapa*, *Brassica juncea*, and *Brassica campestris* (Delourme et al., 1998; Verma et al., 2000; Branca, 2008; Chamola et al., 2013; Dong et al., 2013). The successful transfer of this CMS system to *Eruca sativa* has been previously demonstrated by Nothnagel et al. (2013, 2016).

In contrast, no functional system for the control of pollination is available for *D. tenuifolia*. The most promising option to establish male-sterile *D. tenuifolia* lines was to combine the nucleus of wall-rocket via intergeneric hybridisation with the cybrid cytoplasm of a CMS-*B. oleracea*. While interspecific crosses within the genus *Brassica* have been reported frequently (for review, see Nishiyama et al., 1991; Branca, 2008; Ordas and Cartea, 2008; Kaneko et al., 2011), the success rate for intergeneric crosses within the Brassicaceae family is significantly lower. Nonetheless, some hybridisations between *Diplotaxis* species and *Brassica* crops were successful (Hinata and Konno, 1979; Ringdahl et al., 1987; Chatterjee et al., 1988; Delourme et al., 1998; Batra et al., 1990; Nanda Kumar and Shivanna, 1993; Banga et al., 2003; Inomata, 2003, 2005; Garg et al., 2007; Jeong et al., 2009; Fujita et al., 2020). However, all have been conceived to utilise resistance traits detected in *Diplotaxis* for *Brassica* crops or to develop CMS-*Brassic*a using the *Diplotaxis* cytoplasm, rather than developing CMS-*Diplotaxis*.

The present study reports the successful intergeneric hybridisation between *D. tenuifolia* and CMS-*B. oleracea*, the overcoming of crossing barriers, the partial restoration of female fertility, as well as the morphological and histological characterisation of alloplasmic CMS wall-rocket lines ready for hybrid breeding.

## Materials and methods

### Plant material

*D. tenuifolia* L. (2*n* = 2*x* = 22, DD) seeds were obtained from commercial seed traders: cv. ‘Wilde Rauke’ (Dt 01) from International Seeds Processing GmbH, Quedlinburg, Germany and cv. ‘Grazia’ (Dt 02) from Rijk Zwaan Breeding, Fijnaart, The Netherlands. The CMS donor was the cauliflower line BAZOG 4 (2*n* = 2*x* = 18, CC) from the working collection of the Julius-Kühn-Institut. This CMS line was developed by protoplast fusion, independent of, but similar to, the procedure described by Pelletier et al. (1983). Whereas the nuclear and plastid genomes descended from *B. oleracea* cv. ‘Korso’, a unique mtDNA rearrangement has been identified (Nothnagel and Budahn, 1994), making it distinguishable not only from the original Ogura mtDNA of *R. sativus* landrace but also from the mtDNA of the Ogu-INRA^®^ CMS system. In detail, BAZOG 4 was described by Nothnagel et al. (2016). Parental plants, hybrids and backcross plants were cultivated in 9 to 16 cm plastic pots within a sand–humus soil mixture (v/v 3:1) under greenhouse conditions (approximately 20/15 °C D/N, 16 h during winter season with additional light [PAR 10,000 lm/m^2^]).

### Crosses and ovary culture

The initial crosses CMS-*B. oleracea* (BAZOG 4) × *D. tenuifolia*, as well as the recurrent backcrosses, were performed manually under insect-protected greenhouse conditions. Siliques with visible seed development were harvested approximately 2 weeks after pollination and were surface sterilised (3% NaClO for 15 min, deionised water for 1 min). Developed ovaries were prepared and cultured in Petri dishes on MS medium (Murashige and Skoog, 1962) containing MS micro- and macroelements, including vitamins (4.4 g/L; Duchefa, Haarlem, The Netherlands), 7.5 g/L Bacto-Agar (Becton Dickinson, Heidelberg, Germany), 30 g/L saccharose, with the pH adjusted to 5.9–6.1 before autoclaving. Petri dishes were placed in a culture chamber at 25/20°C D/N and a photoperiod of 16 h light (PAR 10,000 lm/m^2^). In cases of embryo and shoot development, subculture and shoot and root induction were performed on MS medium + 0.2 ml/L NAA. Ovaries that developed only callus were discarded. Established *in vitro* plants were transferred to plastic pots with sandy-humus soil (v/v 3:1) and cultured in a climatic chamber and later in the greenhouse. Established hybrid plants were multiplied by shoot cuttings directly in soil.

### Polyploidisation

Cloned F_1_ plants were treated with a 0.2% colchicine solution by drop inoculation on the secondary shoot meristem and incubated for 3 days. Approximately 4 weeks later, the putative polyploid shoots were cut and rooted in soil. Genome doubling was investigated by flow cytometry in relation to the F_1_ plants.

### Flow cytometry

Fresh mature leaf material (approximately 25 mm^2^) was chopped with a razor blade in 500 µl of nucleic extraction buffer from the CyStain^®^ PI absolute P Kit (Sysmex, Kobe, Japan) and stained by adding 1 ml of the corresponding staining buffer, CyStain™ PI absolute P (Sysmex), supplemented with 75 µg/ml propidium iodide (Sigma-Aldrich, Taufkirchen, Germany) and 3 µg/ml RNase A (Serva, Heidelberg, Germany). Diploid *R. sativus* (2*n* = 18) was used as an internal reference standard with 2C = 1.1 pg (Bennett and Leitch, 1995). The suspension was filtered through a 35-µm Cell-Strainer Cap (Becton, Dickinson and Company, BD Biosciences, San Jose, CA, USA) and immediately analysed after staining using a FACS Calibur™ (BD Biosciences). For each plant, three independent samples were measured (with at least 5,000 recorded events per measurement). For 2C DNA content, the mean peak positions of the internal reference standard and *Diplotaxis* sample were analysed, supported by the analysis software BD CellQuest Pro (version 5.2.1) or CytExpert 2.3 (Beckman Coulter, Brea, CA, USA). The peak quality was assessed according to the coefficient of variation (CV) value and should always be below 5.0. If this was not the case, the measurement was repeated with newly chopped material. The nuclear DNA contents were calculated as proposed by Doležel et al. (1992): Sample 2C value (DNA pg) = Reference 2C value × sample 2C mean peak position/reference 2C mean peak position. The 1C DNA content was determined by dividing the 2C DNA content by the known ploidy level.

### Chromosome preparation and counting

Actively growing young root tips were collected in distilled water. After 1–1.5 h on ice, the meristem tissue was treated for 2.5 h with 2 mM 8-hydroxyquinoline, followed by fixation in freshly prepared 3:1 ethanol:acetic acid overnight at room temperature and then stored at 4°C. Meristems were rinsed with distilled water for 10 min and then macerated in an enzyme mixture containing 4% cellulase (‘Onozuka R-10’, Duchefa Biochemie, Haarlem, The Netherlands) and 1% pectolyase Y-23 (Seishin Pharmaceutical Co., Tokyo, Japan) in 75 mM KCl and 7.5 mM Na_2_EDTA adjusted to pH 4.0 (Kakeda et al., 1991). Enzymatic digestion was carried out at 37°C for 40 to 50 min. The macerated meristems were washed in distilled water for at least 10 min and squashed in 45% acetic acid. The chromosomes were counted under phase contrast using an Axioskop 2 microscope (Zeiss, Oberkochen, Germany). Images were captured using an AxioCam MRm (Zeiss) camera. For the determination of the chromosome number (2*n*), more than five metaphase cells from at least two individual root tips were analysed.

### GISH/FISH analysis

One microgram of high molecular weight unshared genomic DNA of *D. tenuifolia* was labelled using a 20-µl reaction of the DIG-NICK translation kit (Roche Diagnostics, Mannheim, Germany) for genomic *in situ* hybridisation (GISH). The 5S rDNA, a 117-bp fragment of the gene in soybean described by Gottlob-McHugh et al. (1990), was amplified and labelled by PCR. For blocking, a 30-fold excess of unlabelled *B. oleracea* DNA was used. Each 50 µl hybridisation mix contained 144 ng DIG-labelled *Diplotaxis* DNA, 4,320 ng blocking DNA, 180 ng biotin-labelled 5S rDNA, and 5,000 ng salmon sperm DNA. The indirect FISH protocol was performed according to Schrader et al. (2000).

### Marker analysis

Total DNA was isolated from 0.5 g of young leaf tissue according to the method of Porebski et al. (1997) and adjusted to 10 ng/µl. The double primer RAPD (dpRAPD) analysis followed the protocol of Welsh and McClelland (1991), combining two different Operon decamer primers. The amplification products were separated on 1% agarose gels in 0.5 × TBE buffer. The 100-bp ladder of Thermo Fisher Scientific (Waltham, MA, USA) was used for size determination. The agarose gels were stained with ethidium bromide solution and documented under UV light.

### Determination of flower architecture and seed set characteristics

#### Flower morphology

The flower morphology was compared with that of the parental lines during different developmental stages. The petal form and colour, the shape of the carpel, nectary formation, and the development of anthers were documented using a stereomicroscope.

#### Anther development and male sterility

For comparison of anther development and microsporogenesis, flower siliques (3–6 mm long) were fixed in Zinc Formal-Fixx (Life Science International, Wayne, IL, USA) diluted with water (1:4), dehydrated in a graded ethanol series, and embedded in Historesin (Leica, Heidelberg, Germany). Semi-thin sections (4 µm) were cut from the polymerised blocks with an RM2155 microtome (Leica, Wetzlar, Germany), stained with 0.01% toluidine blue, and analysed with a Nikon 90i microscope equipped with an M5 digital camera and NIS Elements BR 3.2 software (Nikon, Düsseldorf, Germany).

#### Carpel development and female fertility

Longitudinal sections of carpels were prepared using a scalpel to evaluate the ovaries during the final flower development phase (first day of opened flower). Carpel and stylus length was measured, and the number of normal and disturbed ovaries was counted. The mentioned histological flower sections also enabled a characterisation of the carpel development.

#### Seed set characteristics

BC_5_ to BC_7_ plants were evaluated for their seed set characteristics. Harvested siliques were measured, and the seed number per silique was calculated as a measure of female fertility. Seed characteristics such as shape, size, and colour were determined. Additionally, the percentage of normal, shrunken and dried seeds as well as the number of pregerminated seeds per silique was counted. The germination capacity was calculated as a measure of seed quality.

For estimation of the seed set ability, three to five plants of four different BC_7_ lines were placed in separate gauze cages together with four plants of the *D. tenuifolia* parent as pollinator. During the flowering period, flies (‘Red Pinky’, commercial Grebenstein Angelsport GmbH, Giesen, Germany) were used for pollination. Every 20 mature siliques per BC_7_ and Dt 01 plant were harvested, measured, and calculated for seed quantity and quality.

#### Seed quality characterisation by 3D X-ray CT

Individual seeds were placed into X-ray transparent discs (phenoLytics GmbH, Timmendorfer Strand, Germany) for phenotyping with a 3D X-ray CT system using the phenoCheck application of a ST Scan Compact machine (SeedTelligence, Gravenzande, The Netherlands). The underlying technology was described previously (Porsch, 2020) and used routinely for seed phenotyping in industrial settings. X-ray scans were carried out at 28 µm volumetric pixel (voxel) resolution. Virtual 2D cross-sections from the 3D X-ray reconstructions were generated in the maximal XY dimension for each seed to provide a first visual assessment of internal seed quality and morphology.

Seed-level (external) and seed-organ-level (internal) parameters were automatically extracted by applying an image processing pipeline that provided 3D measurement data for each individual seed (provided by phenoLytics GmbH). For organ parameters, the embryo, pericarp, and empty space in the seed were segmented and measured, whilst additional relative values such as specific weight and relative embryo volume (embryo volume relative to the seed volume) were calculated based on these extracted values.

Seeds were clustered (phenotypic cluster) initially visually using 3D X-ray reconstructions for similar seed quality characteristics. This clustering was refined by supervised and unsupervised analyses based on the extracted measurement values. Hierarchical clustering was performed to group seeds based on the combined seed-level and organ-level measurement values per seed. To assess the similarity between the hierarchical cluster and defined phenotypic cluster, the Adjusted Rand Index (ARI) was calculated. In addition, a supervised machine learning model (Light Gradient Boosting Machine [LightGBM]) was trained to predict the defined cluster based on seed-level and organ-level measurement values per seed. Model performance was evaluated using standard metrics, including accuracy. Finally, principal component analysis (PCA) was used to visualise the clustering structure and the agreement between the hierarchical and defined clusters. All analyses were performed in Python (version 3.11.12) using standard scientific computing libraries. Hierarchical clustering was conducted using SciPy (Virtanen et al., 2020), whilst PCA and the ARI were implemented using scikit-learn (Pedregosa et al., 2011). The LightGBM model was implemented using the LightGBM package (Ke et al., 2017).

### Statistical analysis

Descriptive statistics (mean, standard deviation) and one-way analysis of variance (ANOVA) followed by Tukey’s test were performed using Statistica version 7.1 (StatSoft Europe GmbH, Hamburg, Germany). Where ANOVA yielded significant *F*-values, means were compared using Tukey’s procedure on a significance level of *α* = 0.05 marked with small letters in the tables. Box plots were generated in Excel (Microsoft Office Standard Package, 2016).

## Results

### Intergeneric hybridisation

More than 300 initial crosses BAZOG 4 × Dt 01 have been carried out, but most of the juvenile siliques were aborted within 10 days after pollination. In approximately 2% of the siliques, single ovaries developed seed-like structures. However, all of these collapsed during the juvenile stage, and no mature seeds were harvested. Therefore, developing ovaries were prepared from the siliques and rescued *in vitro*. Eleven prepared ovaries were established *in vitro* to finally obtain two F_1_ plants from independent crosses.

**Figure 1:**
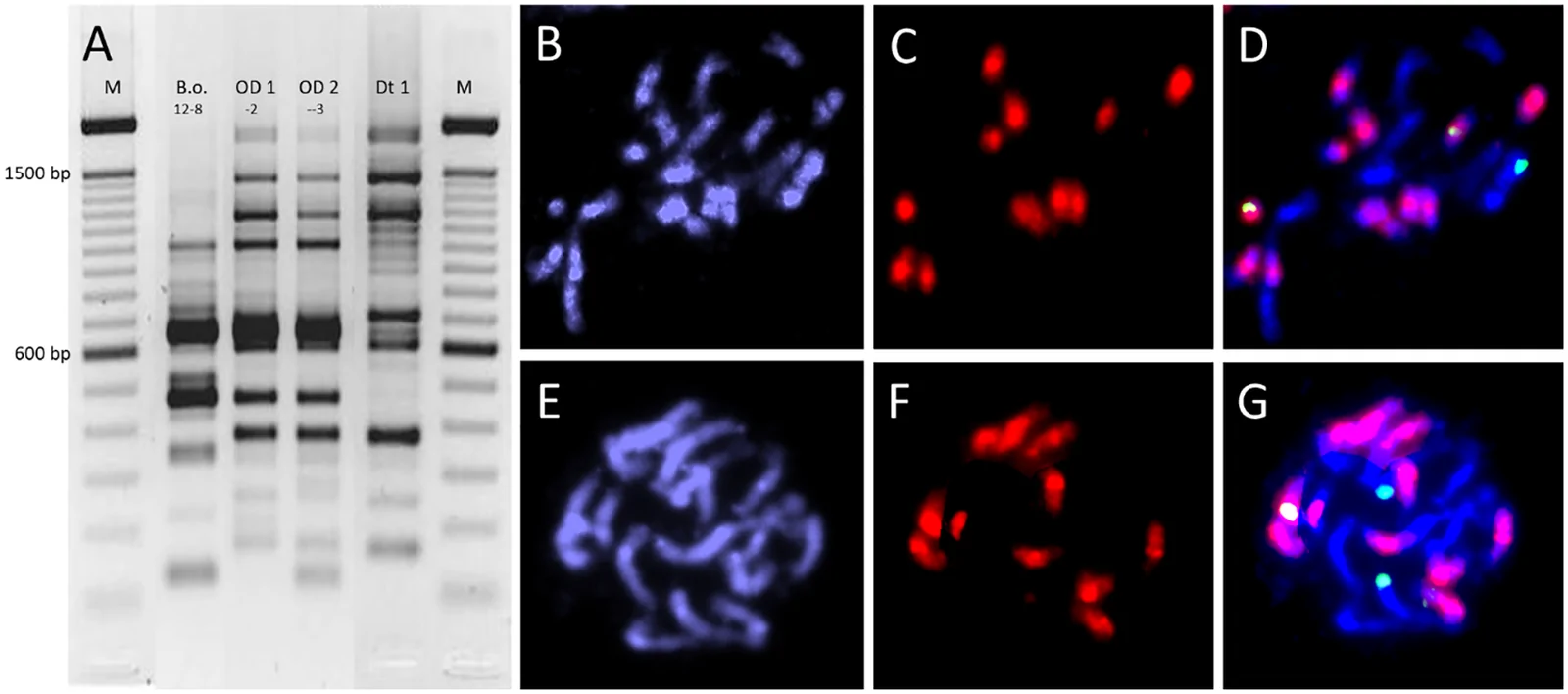
Confirmation of the hybrid character of the intergeneric hybrid BAZOG 4 × Dt 01. **(A)** Test for hybrid status using dpRAPD primer combination OPA17 + OPA18; lanes 1 and 6: 100 bp ladder; lane 2: BAZOG 4; lane 3: F_1_ OD1–2; lane 4: F_1_ OD2–3; lane 5: Dt 01. **(B–D)** Test for hybrid status using GISH and FISH analyses. **(B–D)** OD1–2. **(E–G)** OD23. **(B, E)** OD20 (11 *D. tenuifolia* and nine *B. oleracea*) DAPI-stained mitotic chromosomes (blue). **(C, F)** Eleven GISH-labelled *D. tenuifolia* chromosomes (PI; red). **(D–G)** Overlay of the DAPI and PI signals with light yellow signals from FISH-labelled 5S rDNA (2 × 5S rDNA signals on *D. tenuifolia* chromosomes/1 signal on a *B. olerace*a chromosome).

After *in vitro* regeneration of plants and transfer to soil, the hybrid character of the individuals was verified by molecular markers. The two F_1_ plants showed slight differences in their banding patterns (**Figure 1A**). Additionally, the chromosome number was determined, and the chromosome sets were discriminated by genomic (GISH) and gene-specific (FISH) *in situ* hybridisation. For the two F_1_ hybrids, the expected number of 11 *D. tenuifolia* and nine *B. oleracea* chromosomes was determined (**Figures 1B–G**).

### Recurrent backcrossing

**Figure 2:**
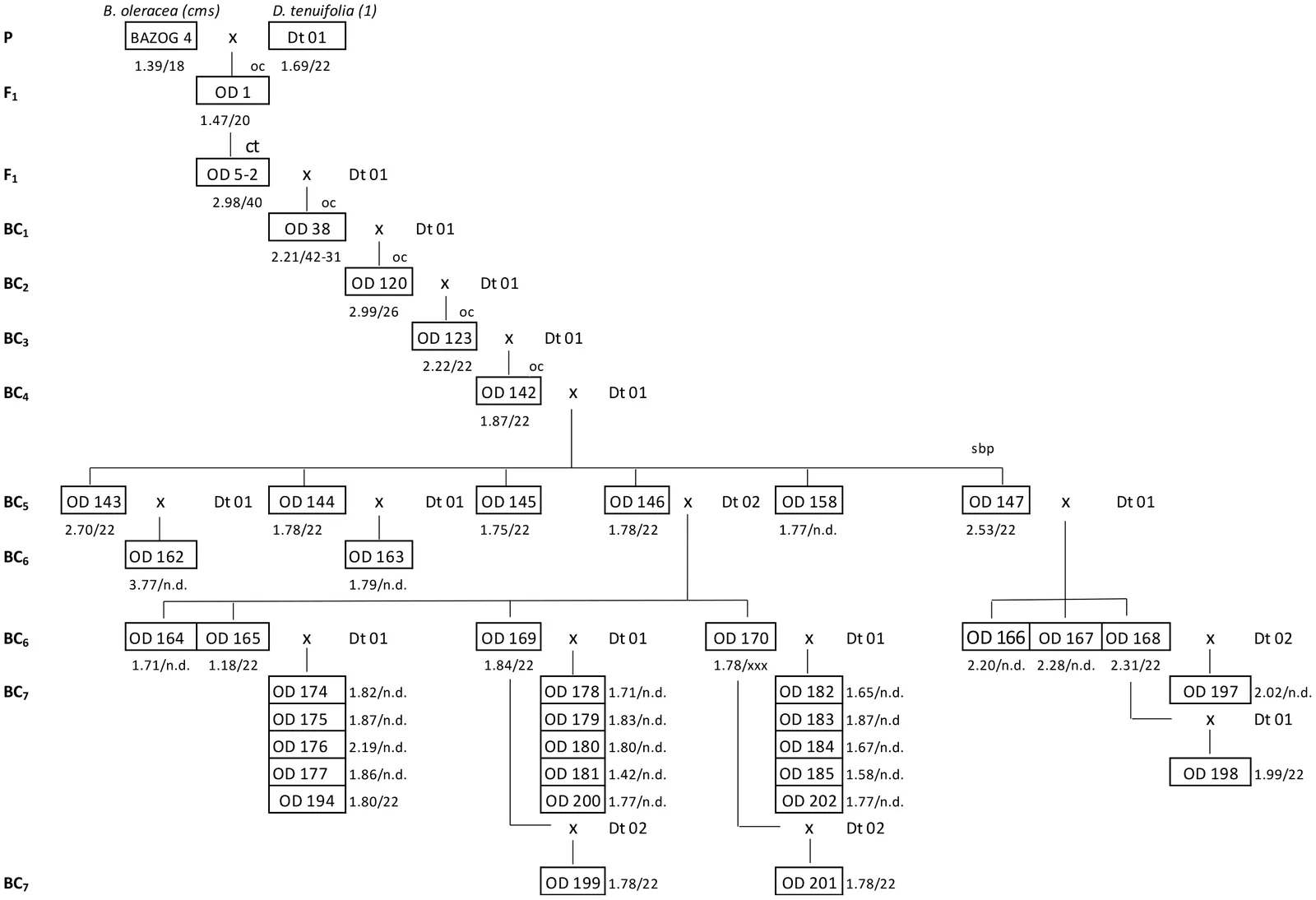
Crossing pedigree for the development of CMS wall-rocket lines. The pedigrees of CMS lines derived from *D. tenuifolia* from the initial intergeneric cross to the BC_7_ generation are shown as an extract from the entire crossbreeding programme. Under or to the right of the boxes with plant ID are listed the C2 DNA value in picograms estimated by flow cytometry and the chromosome number counted in root tip metaphase cells. oc, ovary cultivation; ct, colchicine treatment; sbp, seed-borne progeny; n.d., not determined.

**Figure 3:**
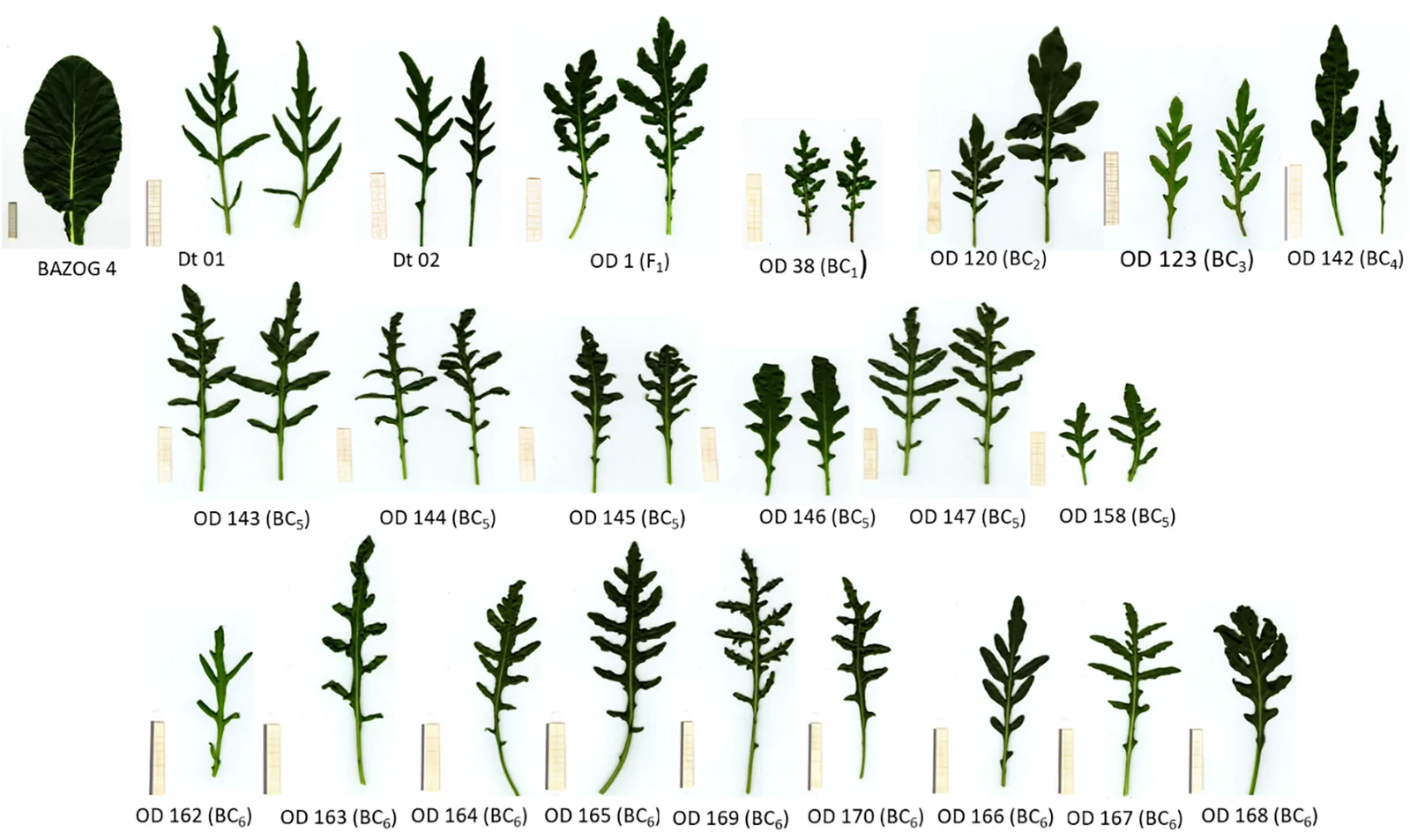
Representative leaf morphology of CMS-*B. oleracea*, *D. tenuifolia*, their F_1_, and the OD hybrids of the backcross generations BC_1_ to BC_6_. Bar = 5 cm.

**Figure 4:**
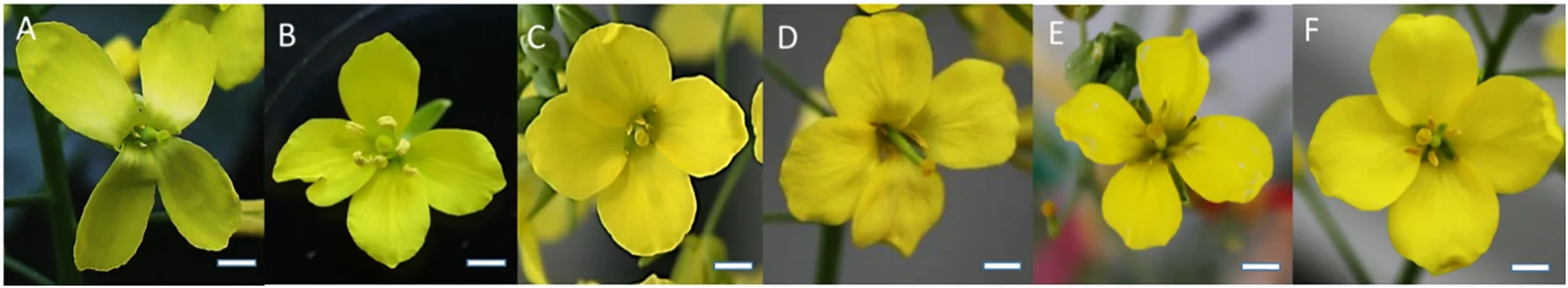
Flower architecture of **(A)** male-sterile *B. oleracea* (BAZOG 4), **(B)** male-fertile *D. tenuifolia* (Dt 01), **(C)** male-sterile OD hybrid OD 1 (F_1_), **(D)** male-sterile hybrid OD 38 (BC_1_), **(E)** male-sterile hybrid OD 142 (BC_4_), and **(F)** OD 199 (BC_7_). Bar = 2 mm.

The pedigree of the described CMS wall rocket material was summarised in **Figure 2**. The two F_1_ plants obtained from the primary cross showed similar growth and leaf morphology as the *Diplotaxis* parent (**Figure 3**). Also, inflorescence and flower characteristics more closely resembled the *Diplotaxis* parent (**Figure 2**). The flowers of the hybrids were male sterile but showed a normally developed pistil and carpel. Both plants showed no seed set after pollination with the *Diplotaxis* parent. Therefore, rooted shoot cuttings of the F_1_ plants were treated with colchicine to obtain tetraploid plants (2*n* = 4*x* = 40; CCDD). Altogether 16 colchicine-treated clone plants (F_1_) were generated. Five of these, which were confirmed by flow cytometry to have a doubled genome size, were backcrossed with the *D. tenuifolia* parent (**Table 1**). Ovary culture was necessary to generate 15 BC_1_ plants from 97 putative crossing events. These plants showed a morphology mostly similar to that of the *Diplotaxis* parent and expressed male sterility (**Figures 3**, **4**). The chromosome number varied between 35 and 50. Further backcrosses using *D. tenuifolia* as the pollen parent were successful for BC_1_ plants with 42, 46, and 50 chromosomes. All 32 resulting BC_2_ plants were male sterile, and most of them were morphologically highly similar to the *Diplotaxis* parent. In crosses of BC_3_ plants, the female fertility was still unchanged compared with BC_1_ and BC_2_. Two BC_3_ plants were successfully backcrossed, and four BC_4_ plants were generated in the described manner via ovary culture. During backcrosses of the four BC_4_ plants, one individual plant (OD 142; 1.97 pg) developed siliques with a total of six mature seeds. Starting from the BC_5_ plants, grown from these six seeds, no further ovary culture was necessary, so the effort required to produce the next generation became significantly lower (**Table 1**). In rare cases, plants of generations BC_1_, BC_2_, BC_5_, and BC_6_ showed disturbances in flower development, e.g., altered flower organ structure or carpel abnormalities, and were therefore discarded (**Supplementary Figure 1**).

**Table 1:** Overview of the backcross programme, ovary rescue, *in vitro* regeneration, and seed harvest.

Crosses	Plants crossed	Flowers crossed	Siliques developed		Seed setns	Ovaries prepared	In vitro		In vitro plants	In vivo plants	
			n	%			regenerates	%		n	Gen.
*Brassica x Diplotaxis* (Dt 01)	1 x 1	222	4	1.8	0	11	2	18.2	2	2	F_1_
F_1_ x *Dt 01*	2	1619	461	28.5	0	408	97	23.8	15	15	BC_1_
BC_1_ x *Dt 01*	15	2122	634	29.9	0	2475	170	6.9	43	32	BC_2_
BC_2_ x *Dt 01*	60	2220	1216	54.8	0	3003	44	1.5	18	16	BC_3_
BC_3_ x *Dt 01*	15	590	489	82.9	0	2450	37	1.5	4	4	BC_4_
BC_4_ x *Dt 01*	1	71	26	36.6	6	316	117	37.0	10	6 sb	BC_5_
BC_5_ x *Dt 01*	6	257	238	92.6	792					25 *^sb^*	BC_6_
BC_6_ x *Dt 01*	9	429	340	79.3	1184					193 ^*sb*^	BC_7_
BC_7_ x *Dt 01*	12	518	456	88.1	3509						BC_8_
sum		8050	3863			8663	467		92	69/224 ^*sb*^	

*ns*, normal seeds; *sb*, seed borne.

### Flower architecture and male sterility

**Figure 5:**
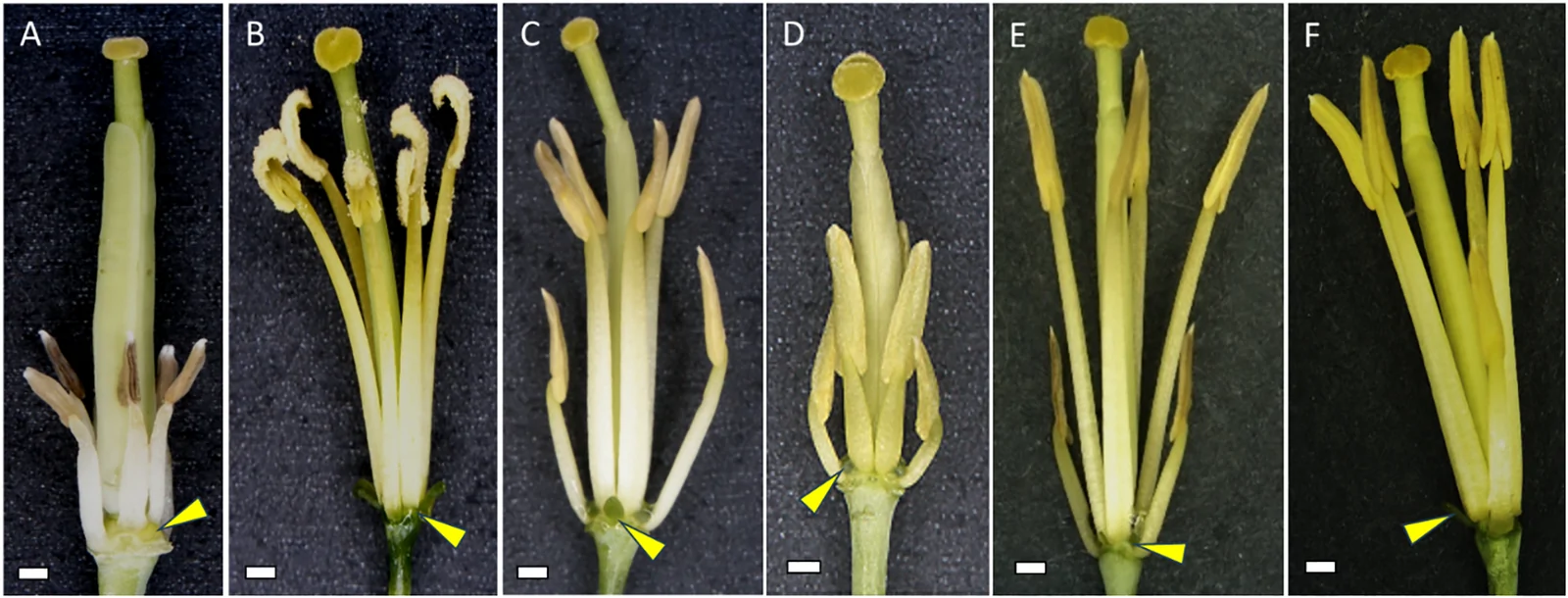
Prepared flowers with anthers and carpels. **(A)** Male-sterile *B. oleracea* parent (BAZOG 4). **(B)** Male-fertile *D. tenuifolia* parent (Dt 01). **(C)** Male-sterile OD 1 (F_1_). **(D)** Male-sterile OD 38 (BC_1_). **(E)** Male sterile OD 142 (BC_4_). **(F)** Male-sterile OD 199 (BC_7_). Arrows mark nectaries. Bar = 1 mm.

The F_1_ and backcross plants of the BC_1_ to BC_7_ generations expressed dark yellow petals, as did both parental lines (**Figure 4**). The flower morphology was highly similar to that of the *Diplotaxis* parent (**Figure 4**). Normal carpels and nectaries were observed in all these plants (**Figure 5**). Variation was recognised in the length of the anthers, despite the general expression of four long and two short filaments. The anther development was normal during the early organ development stage (**Figure 5**).

**Figure 6:**
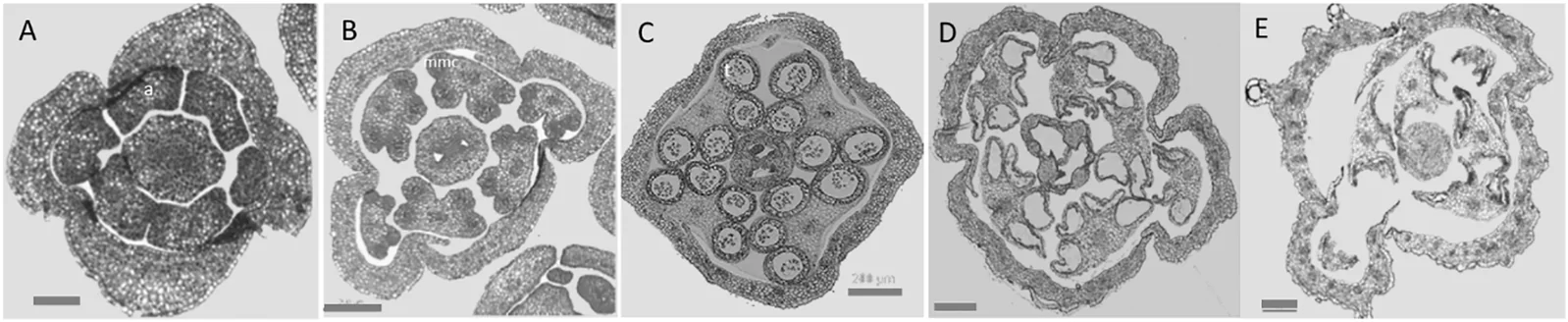
Transverse sections illustrating the development of juvenile flowers of OD 120 (BC_2_) during anthesis. **(A)** Microsporangium stage of six anthers (a). **(B)** Anthers in the early microspore mother cell (mmc) stage. **(C)** Late tetrad (t) stage of meiosis, with partially collapsed microspores. **(D)** Collapsed microspores and imploding of anthers. **(E)** Completely shrunken anthers. Bar = 200 µm.

**Figure 7:**
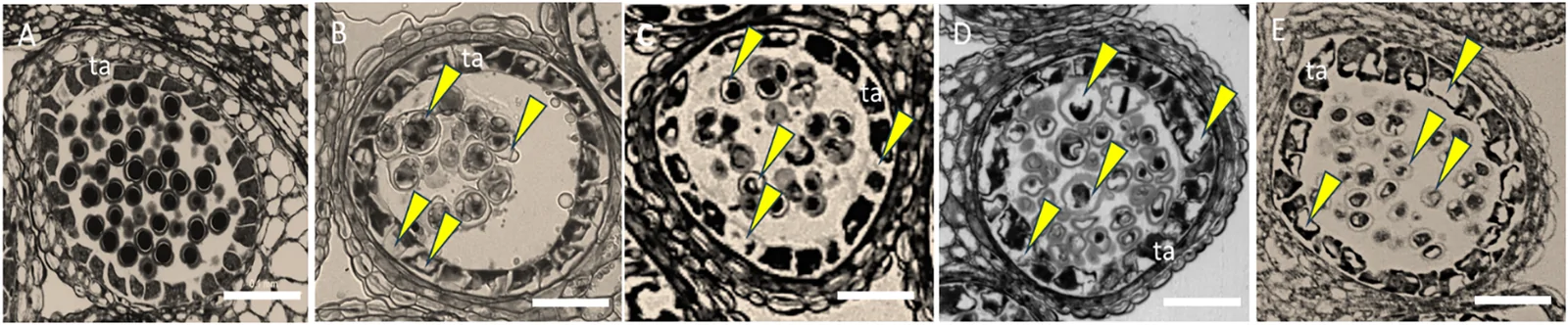
Transverse sections of anther locules during late meiosis. **(A)** Locule of male-fertile *D. tenuifolia* with microspores surrounded by the exine wall. **(B–E)** Locules of male-sterile OD plants during the late tetrad and early microspore stages, with collapsing tetrads and microspores as well as a partially vacuolised or disturbed tapetum layer. **(B)** OD 38 (BC_1_). **(C)** OD 120 (BC_2_). **(D)** OD 123 (BC_3_). **(E)** OD 199 (BC_7_). Arrows mark disturbed microspores and tapetum cells (ta). Bar = 100 µm.

Each flower showed six anthers with typical butterfly symmetry and normal thecae and locule development. Histomorphological analyses revealed anther breakdown starting in early stages of meiosis (anaphase I), analogous to the CMS-*B. oleracea* control (not shown). A progressive vacuolisation of the tapetum layer and an impaired microspore development was observed up to the tetrad stage. At the tetrad stage, locule collapse also starts, which is completed before flower bud opening (**Figures 6**, **7**). Pollen grain spilling was not observed at any time.

### Carpel and ovary development

**Figure 8:**
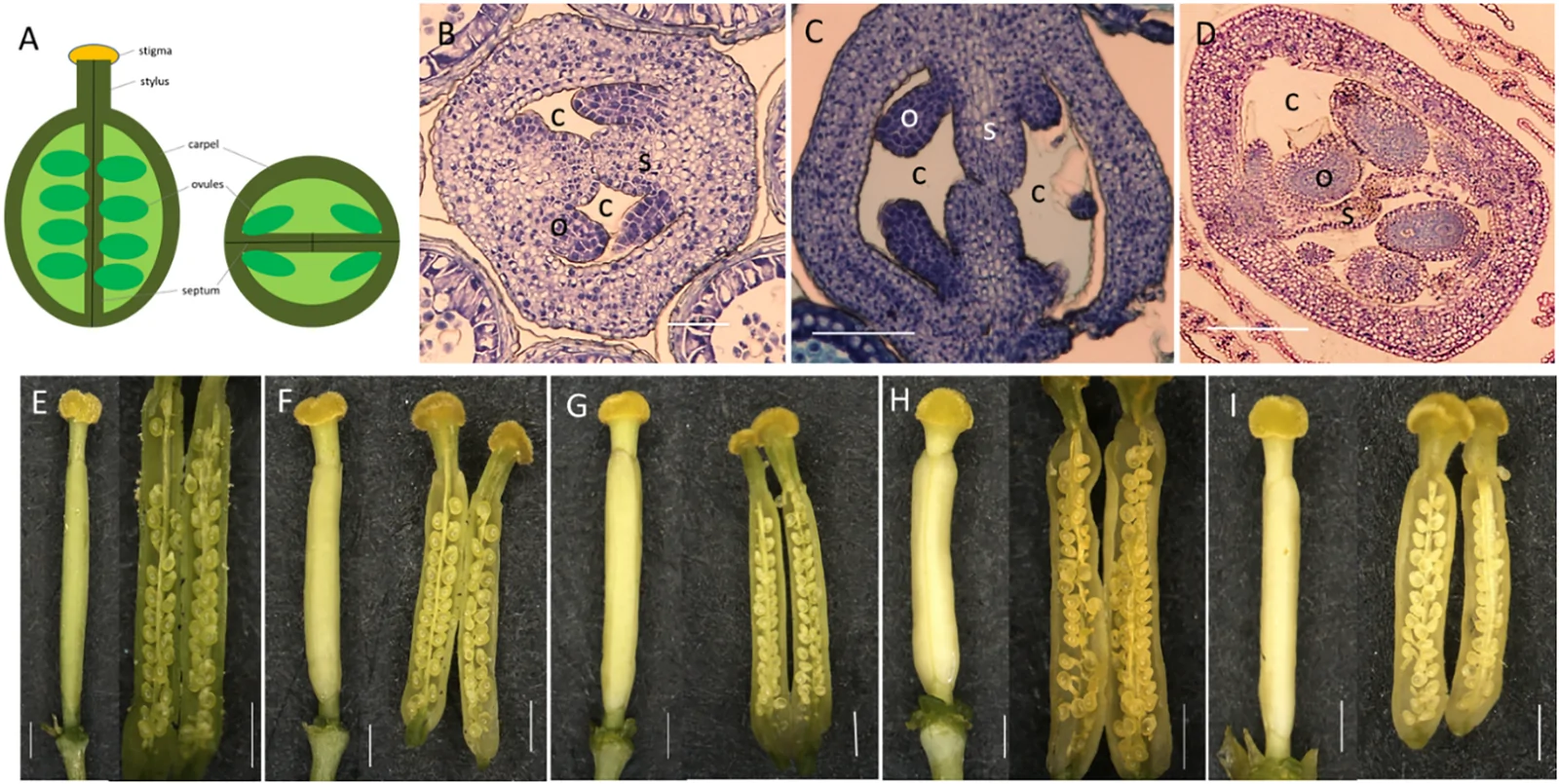
Stigma, stylus, and carpel development of selected OD hybrids compared to *D. tenuifolia* (early flowering stage before pollination). **(A)** Scheme of the carpel of the Brassicaceae family with two fruit compartments, separated by a septum, and two rows of ovaries per compartment (left: horizontal, right: vertical). **(B–D)** Transverse sections of juvenile carpels of OD 38 (BC_1_) **(B)**, OD 120 (BC_2_) **(C)**, and OD 146 (BC_5_) **(D)** showing two compartments (c), a septum (s), and two ovaries in each compartment. **(E–I)** Carpel architecture of *D. tenuifolia* **(E)** compared with **(F)** OD 38 (BC_1_), **(G)** OD 120 (BC_2_), **(H)** OD 146 (BC_5_), and **(I)** OD 165 (BC_6_). Bar = 200 µm **(B–D)** and 1 mm **(E–I)**. (Please compare the carpel architecture of *B. oleracea* in **Figure 5**).

Since the hybrids and backcross plants up to BC_4_ did not set seed, the development of the carpels and ovaries was examined to exclude morphostructural disorders as a cause. No significant differences were found in carpel morphology compared to the *Diplotaxis* parent. Each carpel showed two pseudo-chambers (separated by a septum), with two rows of ovaries in each. The carpels of the F_1_ and BC_1_ plants exhibited slightly curved carpels, whilst those of later backcross generations were as upright as those of *Diplotaxis*. The carpel length of the hybrids was similar to that of *Diplotaxis* or slightly shorter (**Table 2**; **Figure 8**).

**Table 2:** Carpel and ovary characteristics at late pod stage (before pollination).

Gen.	ID	Flowers	Carpel length (mm)	Carpel shape	Ovaries per silique	Normal ovaries	Normal ovaries	Smaller ovaries	Deformed ovaries
		n	Mean ± SD		Mean ± SD	Mean ± SD	%	Mean ± SD	Mean ± SD
Pop	Dt 01	4	10.47 ± 1.26 a	Erect	61.00 ± 8.49 b	56.00 ± 7.62 c	91.86	5.00 ± 1.41 b	0.00 ± 0.00 n.d.
Pop	Dt 02	4	8.31 ± 1.15 c	Erect	53.75 ± 4.57 d	51.25 ± 5.38 d	95.35	2.50 ± 3.11 c	0.00 ± 0.00 n.d.
F1	OD 1	20	9.72 ± 1.28 b	Slightly curved	50.55 ± 3.98 d	49.75 ± 3.74 d	98.46	0.70 ± 1.03 e	0.01 ± 0.31 d
BC1	OD 38	4	7.61 ± 1.16 d	Slightly curved	45.25 ± 6.29 e	35.25 ± 8.38 e	77.34	8.00 ± 3.56 a	2.00 ± 1.63 a
BC2	OD 120	4	10.06 ± 0.23 a	Erect	56.50 ± 9.85 d	53.75 ± 9.22 d	95.20	2.75 ± 1.71 c	0.00 ± 0.00 n.d.
BC4	OD 142	4	8.42 ± 0.38 b	Erect	50.50 ± 3.42 d	50.00 ± 2.58 d	99.09	0.50 ± 1.00 e	0.00 ± 0.00 n.d.
BC5	OD 146	4	7.84 ± 0.79 cd	Erect	66.50 ± 16.70 b	63.00 ± 14.49 b	95.19	2.00 ± 0.82 c	1.50 ± 3.00 b
BC5	OD 147	4	10.21 ± 0.81 a	Erect	88.50 ± 4.20 a	83.50 ± 2.65 a	94.41	4.75 ± 1.50 b	0.25 ± 0.50 c
BC6	OD 165	4	7.82 ± 1.50 cd	Erect	59.50 ± 10.54 c	54.75 ± 7.54 d	92.46	4.50 ± 3.32 b	0.25 ± 0.50 c
BC7	OD 207	4	8.13 ± 0.94 c	Erect	61.25 ± 6.08 b	59.50 ± 6.95 c	97.03	1.75 ± 1.50 d	0.00 ± 0.00 n.d.

Different letters indicate significant differences at *p* < 0.05.

The number of developed ovaries was comparable to that of *Diplotaxis*, although it was significantly lower for OD 38 (BC_1_) and significantly higher for OD 147 (BC_5_). The proportion of smaller or deformed ovaries was significantly increased in OD 38 (BC_1_). The proportion in the other hybrid plants was very similar to that of *Diplotaxis* and was negligible overall. A significant ratio of smaller or deformed ovaries was observed in the BC_1_ and BC_2_ generations (**Table 2**; **Figure 8**). In the crosses from the F_1_ to BC_3_ generations, most ovaries dried out and shriveled within a few days after pollination (**Table 1**).

### Silique and seed set after hand pollination

The silique set after hand pollination in the BC_5_ to BC_7_ generations ranged between 79% and 93%, comparable to the *Diplotaxis* silique set. In contrast, the number of germinating seeds per silique was by far lower than that of *Diplotaxis* (**Table 3**). The data for each individual progeny are shown in **Supplementary Tables 1**-**3**.

**Table 3:** Summary of seed quantity and quality harvested from BC_5_ to BC_7_ plants.

Plant ID	Gen.	Plants	Flowers crossed	No of siliques harvested		Seeds per silique	Seed fractions harvested in total (cluster)								
							Total	Normal (A)		Pregerminated (B)		Shrunken (C)		Dried (D)	
		n^a^	n	n	%		n	n	%	n	%	n	%	n	%
OD 143–158	BC_5_	6	257	238	92.60	24.64	5,864	792	13.51	90	1.56	3,075	52.44	1,907	32.52
OD 162–170	BC_6_	9	429	340	79.25	26.31	8,946	1,184	13.23	70	0.78	5,214	58.28	2,478	27.69
OD 174–185	BC_7_	12	559	490	87.67	38.04	18,641	3,509	18.82	260	1.39	12,408	66.56	3,782	20.29
OD 194–201	BC_7_ op	16		320		33.18	10,618	1,241	11.69	132	1.24	6,114	57.58	3,031	28.46
Dt 1	Dt 1 op	4		83		37.73	3,132	2,394	76.44	35	1.12	165	5.37	538	17.18

^a^ Data for the individual plants are given in **Supplementary Tables 1**-**3**; **Table 2**.

*op*, open-pollinated (flies).

**Figure 9:**
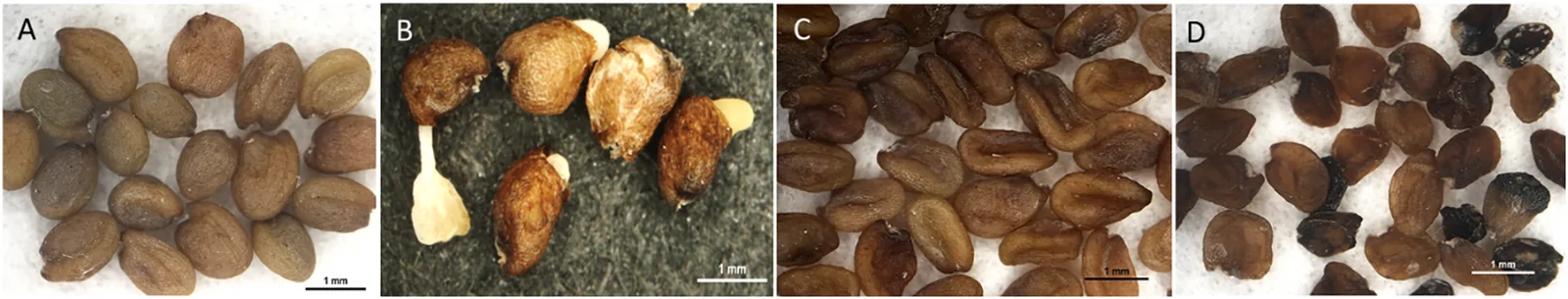
Visually determined seed clusters. **(A)** Normal seeds with high germination capacity. **(B)** Pregerminated seeds with burst seed coats or the beginning of germination within the mature silique. **(C)** Shrunken seeds showing an extremely low germination capacity. **(D)** Totally dried seeds without germination capacity (probably unfertilised or early collapsed ovules). Bar = 1 mm.

For more detailed analysis, the dry seeds harvested from pods of BC_5_ to BC_7_ were separated into four visually distinct fractions. In addition to the cluster of normally developed seeds (cluster A), which strongly resembled the *Diplotaxis* parent, a small fraction of pregerminating seeds (cluster B) was observed. The largest fraction consisted of seeds that shrank significantly during maturation. Visually, a large embryo appeared to be densely covered by the seed coat (cluster C). A fourth fraction consisted of empty seed coats of unfertilised or early collapsed ovules, without any germination capacity (cluster D) (**Figure 9**). The visually classified normal seeds (A) exhibited a medium to high germination rate of 40% to 80%. The pregerminated seed (B) could be partially stimulated to regenerate seedlings when placed on moist filter paper or in soil immediately after harvest. Germination experiments with seeds from fraction C showed only a low germination rate (< 5%) (data not shown).

### Seed set after pollination by flies

In addition to the seed set after hand pollination, seed set was also determined under the more practically relevant conditions of pollination by flies. This was determined for each of three to five individual fly-pollinated plants of the BC_7_ lines OD 194, OD 197, OD 199, and OD 201. Twenty well-developed pods were harvested per plant, the number of seeds was counted, and seed quality, thousand-seed weight (TSW), and germination capacity were assessed (**Table 4**).

**Table 4:** Seed quantity and quality harvested from BC_7_ plants that were open-pollinated by flies within gauze cabins.

ID	Unit	Dt 1	OD 194	OD 197	OD 199	OD 201
Plants	n	4	4	3	5	4
Siliques harvested	n	80	80	60	100	80
Size of siliques						
Length (mm)	Mean ± SD	38.6 ± 4.8 a	25.5 ± 3.8 c	29.1 ± 3.8 b	24.5 ± 4.4 c	24.3 ± 2.7 c
Diameter (mm)	Mean ± SD	2.0 ± 0.3 b	2.1 ± 0,4 b	2.3 ± 0.3 a	2.2 ± 0.3 a	2.3 ± 0.5 a
Seed cluster						
Normal (A)	Total	2394	205	392	331	313
	Mean ± SD	28.8 ± 12.9 a	2.6 ± 2.5 c	6.5 ± 3.5 b	3.3 ± 2.8 c	3.9 ± 2.8 bc
	Min–Max	1–51	0–11	1–10	0–11	0–11
Pregerminated (B)	Total	35	39	40	39	14
	Mean ± SD	0.4 ± 0.9 ab	0.5 ± 0.8 ab	0.7 ± 1.1 a	0.4 ± 0.6 ab	0.2 ± 0.4 b
	Min–Max	0–4	0–4	0–6	0–3	0–2
Shrunken (C)	Total	165	1,673	1,085	2,005	1,351
	Mean ± SD	2.0 ± 2.7 d	20.9 ± 8.9 a	18.1 ± 9.2 ab	20.1 ± 10.1 ab	16.9 ± 9.2 c
	Min–Max	0–13	2–42	2–40	0–47	1–41
Dried (D)	Total	538	653	589	925	964
	Mean ± SD	6.5 ± 5.5 c	8.2 ± 7.2 bc	9.8 ± 6.7 ab	9.3 ± 6.9 abc	12.1 ± 8.9 a
	Min–Max	0–21	0–40	1–35	0–36	0–39
Total		3,132	2,570	2,106	3,300	2,642
	Mean ± SD	37.73 ± 11.8 a	32.12 ± 11.0 b	35.1 ± 9.2 ab	33.0 ± 12.1 b	33.02 ± 9.2 b
	Min–Max	6–67	12–66	19–58	11–65	13–55
% Normal seeds	Mean ± SD	73.8 ± 22.3 a	7.8 ± 6.7 c	18.5 ± 8.7 b	9.9 ± 7.7 c	11.9 ± 8.0 c
	Min–Max	9.5–100.0	0.0–33.3	3.5–37.1	0.0–37.5	0.0–36.8
TSW (g)	Mean ± SD	0.370 ± 0.009 a	0.267 ± 0.023 c	0.310 ± 0.026 b	0.277 ± 0.013 c	0.284 ± 0.021 c
Germination capacity (%)^a^	4 × 20 seeds	93.75 ± 4.78 a	40.00 ± 5.00 b	37.50 ± 3.53 c	48.75 ± 22.86 b	38.75 ± 28.68 c

Mean ± SD and Min–Max are calculated per silique. Thousand-seed weight (TSW; g) was calculated from 3 × 100 seeds and 3 × 65 seeds (OD194). Germination capacity was observed using 4 × 20 seeds each. Different letters indicate significant differences at *p* < 0.05.

^a^ Seeds of cluster A were exclusively tested.

The harvested dry siliques of the OD lines were shorter but, in three cases, thicker than those of the *Diplotaxis* parent. Seed set and fractionation per silique were very similar for the BC_5_ to BC_7_ material (**Supplementary Tables 1**-**3**) regarding total seed set, seed fractions, and the relative proportion of normal seeds. The total number of seeds in the OD lines was slightly lower than that of the Dt lines. The proportion of pregerminated seeds was negligible.

However, substantial differences in the proportion of shrunken and normal seeds were observed. While the *Diplotaxis* parent achieved an average of 73.8% normal seeds per silique, the percentage of normal seeds in the OD lines varied between 7.8% and 18.5%. A maximum of 37% normal seeds was observed in individual OD siliques.

The thousand-seed weight (TSW) was significantly lower than that of *Diplotaxis*, and germination capacity was found to be moderate (**Table 4**).

### 3D X-ray CT phenotyping to identify seed quality issues in OD lines

**Figure 10:**
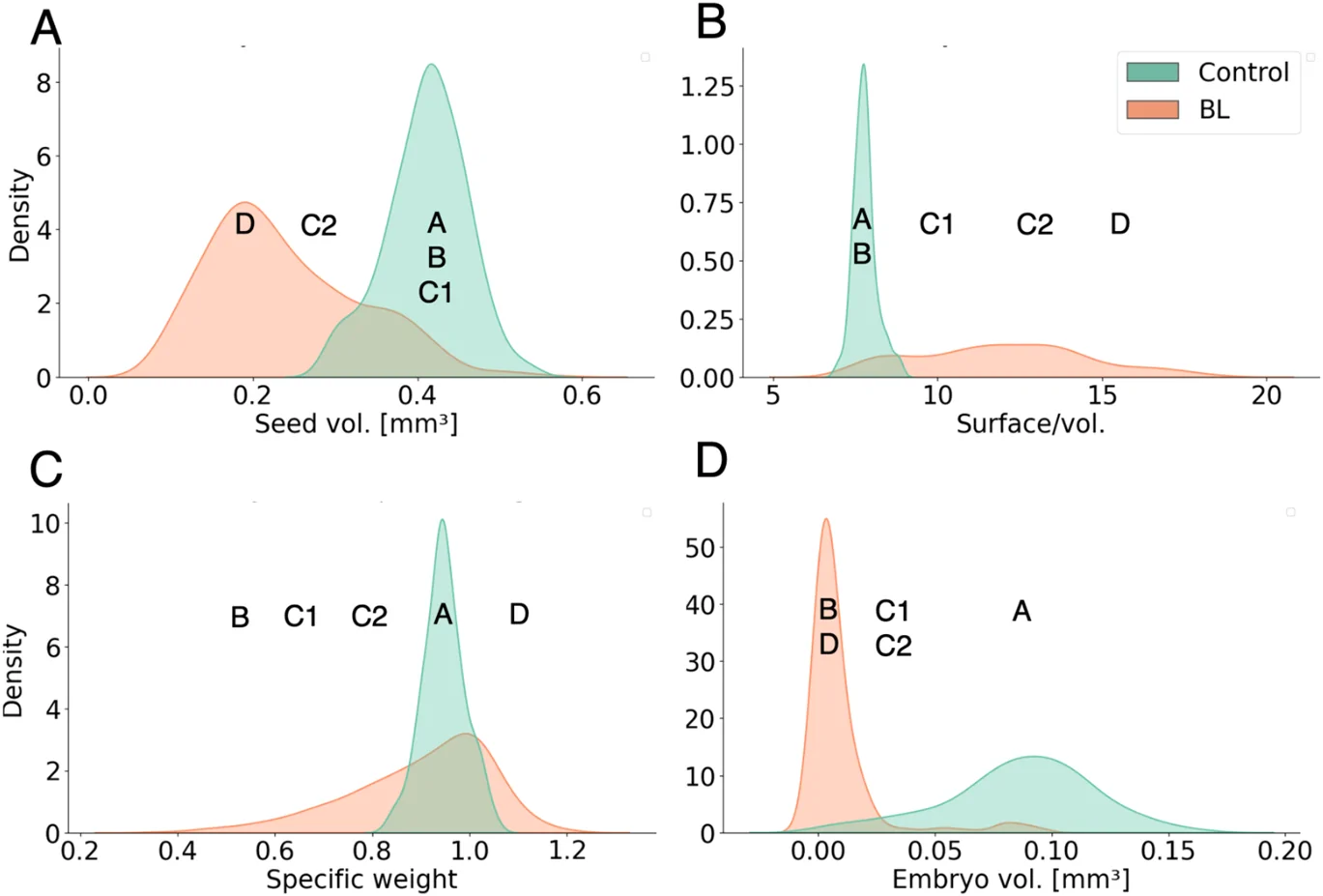
Distribution curves of seed parameters of control vs. breeding lines measured with 3D X-ray CT. Letters along the distribution curves indicate where seeds of each cluster **(A–D)** have their mean peaks.

Representative samples of four BC_7_ OD lines and two control lines were analysed using the 3D X-ray CT phenoCheck method. 3D X-ray CT phenotyping confirmed highly significant differences in seed quality parameters between the control and OD lines, with significant heterogeneity within each population (**Figure 10**; **Supplementary Figure 2**). Seeds of the OD lines had a much higher proportion showing smaller seed volume (**Figure 10A**) and a higher ratio of seed surface area relative to seed volume (*relative seed surface*, **Figure 10B**). Interestingly, seeds with both lower and higher specific weights were observed in the OD lines compared with the control lines (**Figure 10C**).

**Figure 11:**
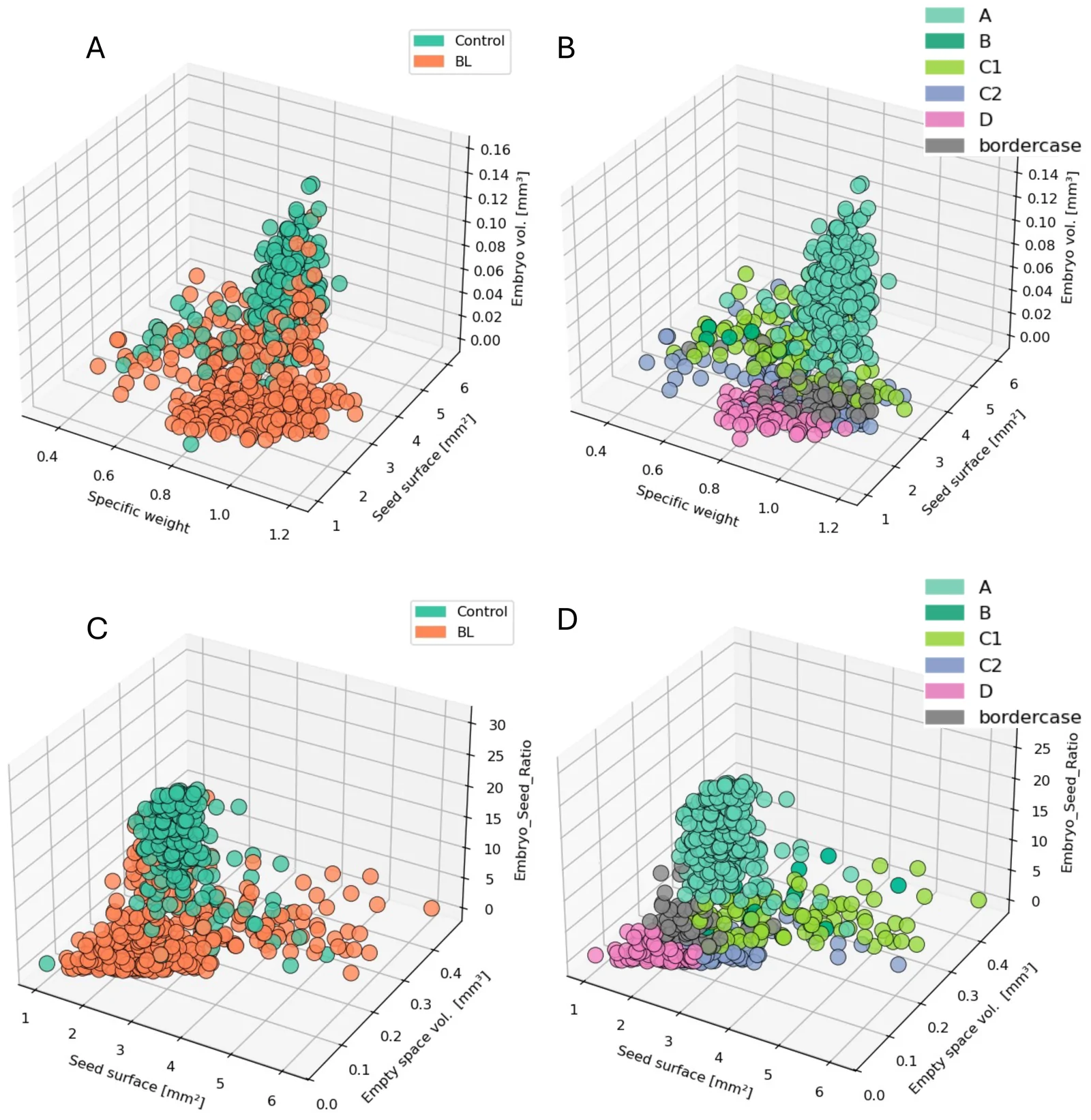
Cluster analyses supporting five distinct clusters. Single seeds plotted by a combination of three seed parameters, coloured according to control vs. breeding lines (BL) **(A, C)** and according to clusters **(B, D)**. Border cases denote seeds that cannot be grouped clearly into any of the clusters.

Regarding internal seed quality parameters, the OD lines displayed, on average, a lower embryo volume, including a lower embryo volume relative to the seed volume (**Figures 10D**, **11A**), and more empty space inside the seed (data not shown). The results thus confirmed a big difference in both seed-level (external) and seed-organ-level (internal) characteristics between the OD lines and the control lines, whilst a small and distinct overlap was observed.

Using 3D X-ray CT imaging and data analysis, we investigated whether the visual discrimination of the four seed quality classes (A–D) based on external characteristics (**Figure 9**) was reflected in internal and quantitative differences in seed quality. Measurements of seed-level parameters, such as seed volume, relative seed surface, and seed-specific weight, broadly confirmed the presence of distinct clusters (**Table 5**; **Figure 11**). However, clustering analysis suggested the presence of a fifth cluster (cluster C2), characterised by a high seed volume, medium relative seed surface, and low specific weight. This clustering was further supported by the internal seed quality parameters, including embryo volume and empty space volume, supporting the presence of five distinct clusters. Both clusters C1 and C2 were found to be distinct based on seed volume, seed surface, and empty space volume (see **Figures 11B, D**; **Supplementary Figure 2**).

**Table 5:** Clustering of seeds based on external and internal seed parameters.

Cluster characterization			Seed characteristics of cluster				
Sub-cluster & characteristics	RGB-Photo of seeds in cluster	3D reconstruction & 2D cross-section for characteristic seeds per cluster	Seed-level			Seed-organ-level	
			Seed volume	Relative seed surface	Seed specific weight	Empty space volume	Embryo volume
Cluster A: Well-developed (normal) Large seeds with well-developed embryo, where enbryo fills the seed cavity fully. Embryo volume and specific weoght high, empty space low.	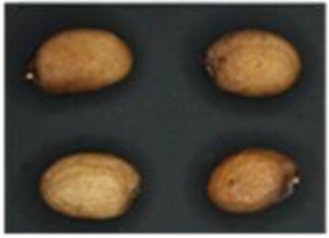	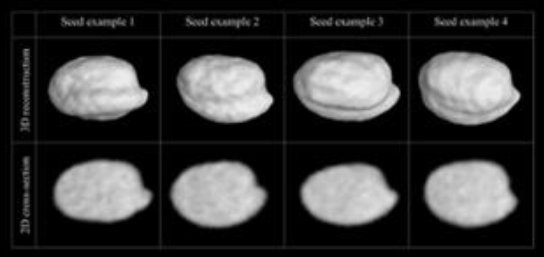	High	Low	High	Very low	Very high
Cluster B: Pre-germinated Large seeds, sometimes with visible pre-sprouting, with only low-density embryo tissue and a high proportion of empty space resulting in a low specific weight	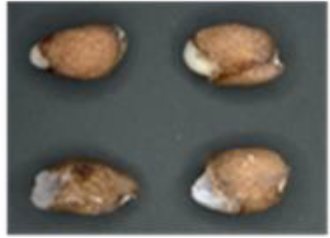	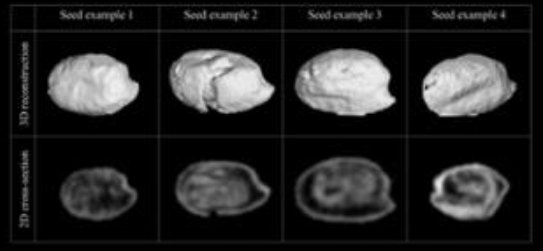	High	Medium/ High	Low	Very high	Very low
Cluster C1: Underdeveloped Large seed with an underdeveloped embryo leading to a high percentage of empty space in the seed. Embryo volume and specific weight are low.	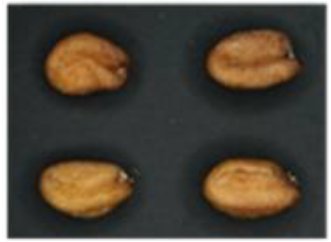	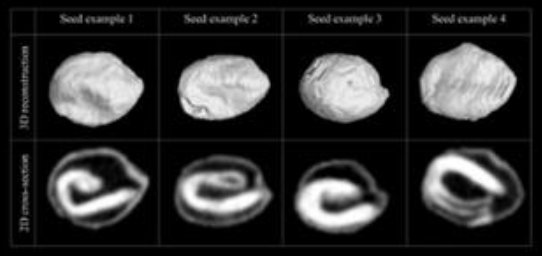	Very high	Medium	Low	High	Low
Cluster C2: Shrunken Small shrunken seeds with an under-developed embryo, tightly covered by pericarp. High specific weoght due to consisting primarily embryo tissue.	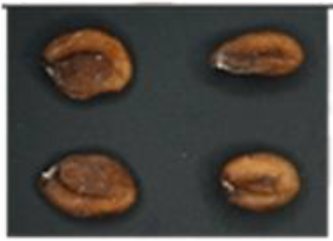	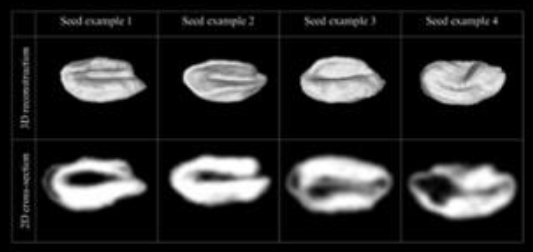	Low-Medium	High	Very high	Low	Low
Cluster D: Dried Small seeds, highest relative seed surface ratio and consist only pericarp, with no or minimal embryo tissue.	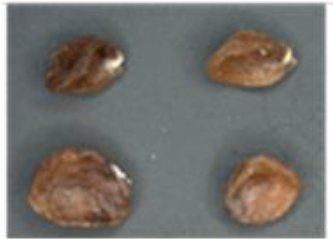	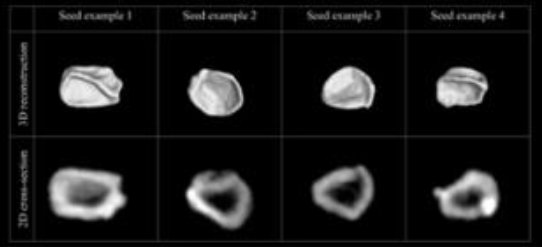	Very low	Very high	Low	Very low	None

The presence and validity of these five defined clusters were confirmed by both hierarchical clustering and a LightGBM-based predictive model. The hierarchical clustering results showed strong agreement with the defined clusters, as visualised in the PCA plots (**Supplementary Figures 3C, D**), and this overlap was quantified by an ARI of 0.61. Using all measured seed quality parameters (seed-level and organ-level), the LightGBM model predicted the five defined clusters with an overall accuracy of 98.5%, with precision values for the individual clusters ranging from 91.7% to 99.3% (**Supplementary Figure 3**). A class of seeds with parameters falling between the distinct clusters was observed (*border case*), with a precision of 98.2% (**Supplementary Figure 3**), which represents a continuous transition between the observed clusters rather than completely distinct groups, as reflected in the distribution curves in **Figure 10**.

The frequency of seeds falling into these defined clusters differed substantially between control lines and OD lines (**Figure 11**), explaining the large deviations in the distribution curves across all seeds (**Figure 10**). Importantly, seeds falling into clusters C2 and D were virtually nonexistent in control lines (percentages < 1%), whilst, taken together, these two clusters made up a majority of seeds in OD lines (see **Figure 11**; **Table 5**). Cluster C1 was also significantly more frequent in OD lines.

Conversely, cluster A of normally developed seeds made up only a minority population in OD lines, whilst it constituted a majority in control lines (see **Figure 11**; **Table 5**). Cluster B of presprouted seeds was found only at low, single-digit percentages across all control and OD lines.

### Plant growth characteristics in generation BC_7_

**Figure 12:**
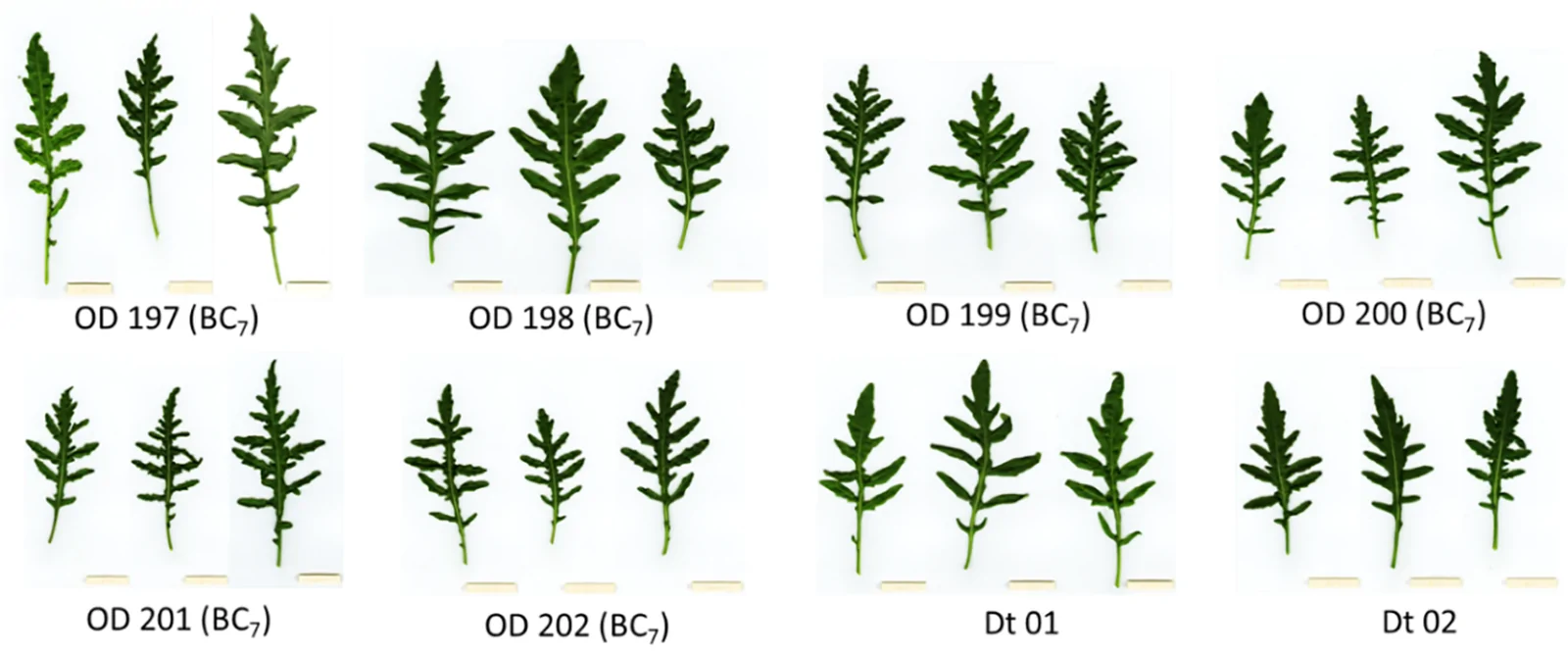
Representative leaf morphology of OD BC_7_ hybrids and the *D. tenuifolia* parent grown in a plastic tunnel at 50 dps. Bar = 5 cm.

**Figure 13:**
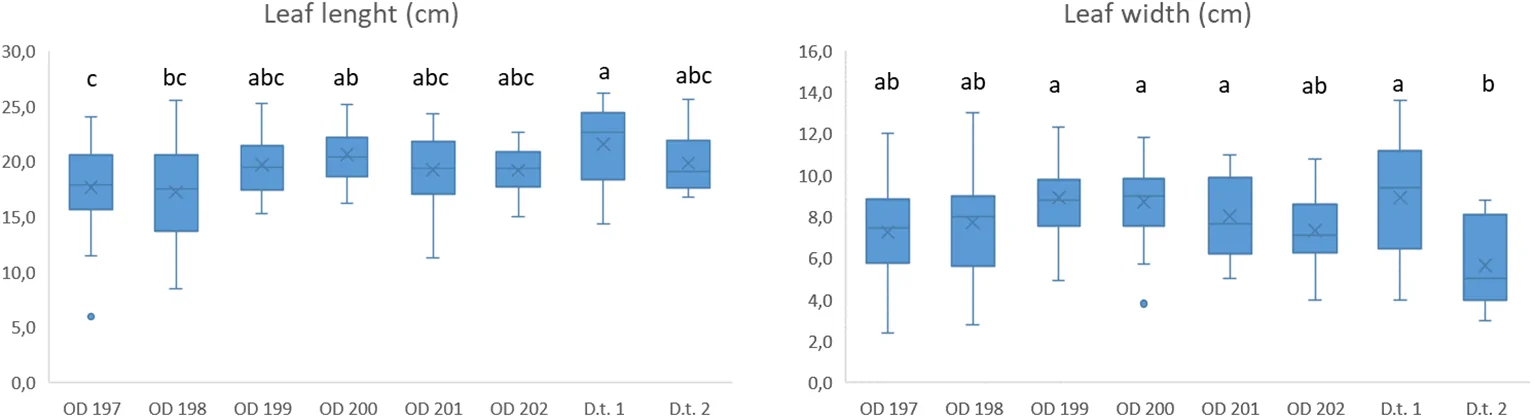
Lamina length and width of OD BC_7_ hybrids and the *Diplotaxis* parents grown in a plastic tunnel. Different letters show significant differences at *p* < 0.05.

In BC_7_, the leaf morphology of the alloplasmic *Diplotaxis* material corresponds already to that of the backcross parent (**Figure 12**). Nevertheless, there are differences between the lines that provide a potential basis for variety breeding. A considerable level of uniformity had already been reached within the lines. Preliminary data collected during the cultivation of BC_7_ (**Supplementary Figure 4**) show that characteristics important for production, such as leaf length and leaf width, are already comparable to those of the *Diplotaxis* parent (**Figure 13**).

## Discussion

To our knowledge, this is the first report on the development of alloplasmic *D. tenuifolia* lines by transferring an alien cytoplasm via intergeneric hybridisation, followed by recurrent backcrosses. The currently obtained BC_7_ lines are diploid with 2*n* = 2*x* = 22 chromosomes, express perfect male sterility, and exhibit a moderate seed set after cross-pollination, suggesting partially restored female fertility. The plants have excellent vigour, and their growth habit corresponds to that of the *D. tenuifolia* parent. Cultivation experiments revealed a yield potential that corresponds to the wall-rocket parent, or even displayed a heterosis effect. Importantly for the breeding process, no further incompatibilities between the CMS-inducing cybrid cytoplasm and the *D. tenuifolia* genome were observed, such as chlorophyll deficiencies or disturbances in flower architecture reported for alloplasmic male-sterile lines in other crops (Sikdar et al., 1990; Prakash et al., 1995; Návratilová et al., 1997; Matsuzawa et al., 1999; Tu et al., 2008).

The cytoplasm source for the development of CMS *D. tenuifolia* breeding lines was the same as that used for the successful development of male-sterile *E. sativa* lines (Nothnagel et al., 2016). Therefore, no general problems were expected. However, seriously reduced female fertility made ovary culture necessary for an additional two backcross generations (BC_4_ for *D. tenuifolia* compared to BC_2_ for *E. sativa*). As the interaction between the *D. tenuifolia* nuclear DNA, chloroplast DNA from *B. oleracea*, and rearranged mitochondrial DNA with parts from *R. sativus*, *B. oleracea*, and *R. sativus*/*B. oleracea* recombined genes (Nothnagel and Budahn, 1994) is highly complex, the reason for the drastically reduced female fertility is still not understood. Nevertheless, in the *D. tenuifolia* backcross programme, one BC_4_ plant (OD 142) also produced some seed-set without ovary culture, making it unnecessary to perform this laborious step for the further backcross generations.

While silique set and silique dimensions are largely comparable to those of the *Diplotaxis* parent, the proportion of normally formed seeds after subsequent backcrossing remained significantly lower (~ 18%). To determine the reason(s) for disturbed seed development and reduced germination capacity, a 3D X-ray CT technique was used. This technique enables a nondestructive insight into the seed and provides a quantitative assessment of embryo development. Cluster A, which comprises normally developed seeds with high germination capacity, constitutes only a small proportion of the OD lines. Heterogeneity of seed quality in cluster A and the presence of pregerminated or otherwise empty seeds in cluster B explain germination rates well below 100% in the control lines. The seeds assigned by visual assessment to fraction C can be divided, according to both seed-level and organ-level characteristics, into the distinct classes C1 and C2. Both classes differ in seed volume, surface, seed-specific weight, and empty space volume. However, the embryo volume is comparable between the two clusters. This suggests that embryogenesis in both C1 and C2 seeds is arrested at a similar stage, whilst in C2 seeds, the seed is simply more dehydrated, leading to a tight wrapping of the pericarp around the embryo. As both clusters occur predominantly or almost exclusively in OD lines, it may be hypothesised that embryogenesis is disturbed due to the breeding process. Alternatively, embryogenesis may simply be delayed and/or more heterogeneous in the breeding lines, leading to a higher proportion of immature seeds at the time of harvest. Similar effects have been reported for carrot seeds, where the maturity of the embryo tissue may be highly dependent on the harvesting time (De Miranda et al., 2017).

Seeds of cluster D were characterised by the smallest volume, the highest surface-to-volume ratio, and a low specific weight. In the pericarp, no or only minimal embryo tissue was detected. This result confirms the presumption made after visual assessment of no fertilisation or the very early death of the pre-embryogenic tissue. As this cluster was observed almost exclusively in breeding lines, inbreeding depression or another cause may be hypothesised.

Only a marginal increase in the proportion of normally developed seeds (cluster A) was observed in the offspring from BC_5_ to BC_7_, from 13.5% to 18.8%. However, a higher proportion of normal seeds of 37% was achieved (**Table 4**; **Supplementary Tables 1**-**3**). A positive selective progress regarding the proportion of pregerminated or burst seeds (B) was achieved in the breeding process. Five OD lines, OD 164 (BC_6_) and OD 182-185 (BC_7_), were selected without burst seeds. This raises the possibility of reducing or even excluding seed clusters B to D by breeding efforts and reaching a germination capacity comparable to the *Diplotaxis* parent.

The presence of clearly underdeveloped seed clusters in breeding lines highlights the need for further research to improve seed quality and germination rates. The established phenotyping of *Diplotaxis* seeds, including the embryo, can predict germination potential. This knowledge can be used to increase the proportion of well-developed seeds by selecting them during the breeding process and/or adjusting maturation times to achieve the highest proportion of normal, viable seeds in the breeding lines, as supported by 3D X-ray CT data. Furthermore, the distinct phenotypic differences in external and internal seed characteristics allow for the targeted sorting of viable seeds with high germination potential (cluster A), thereby enriching the breeding lines with these seeds. This sorting can be performed manually after 3D X-ray CT analysis or using modern sorting equipment and will be the subject of future studies.

The pollination experiment using flies in gauze chambers showed that the CMS lines generally exhibited flowers that were attractive to insects and that insect-mediated pollen transfer occurred as an important prerequisite for seed production within the framework of hybrid breeding. In conclusion, the utilised cybrid cytoplasm proved to be a potential source of CMS for *D. tenuifolia*, and the developed lines are directly applicable to hybrid breeding of rocket salad. However, further work is needed to improve female fertility, including seed set, seed quality, and germination capacity.

## Data Availability

The original contributions presented in the study are included in the article/**Supplementary Material**. Further inquiries can be directed to the corresponding author.

## Supplementary material

Supplementary Table 1 Seed quantity and quality harvested on BC_5_ plants manually backcrossed with *Diplotaxis tenuifolia.*

Supplementary Table 2 Seed quantity and quality harvested on BC_6_ plants manually backcrossed with *Diplotaxis tenuifolia*.

Supplementary Table 3 Seed quantity and quality harvested on BC_7_ plants manually backcrossed with *Diplotaxis tenuifolia.*

Supplementary Figure 1 Flower bud architecture of *Diplotaxis tenuifolia* and bud abnormalities in selected progenies of interspecific hybrids between *Brassica oleracea* x *Diplotaxis tenuifolia* (OD) **(A-C)** normal developed flower buds (control): A – *Diplotaxis tenuifolia;* B – OD 1 (F_1_); C – OD 38 (BC_1_) D-L plants with morphologically abnormalities compared to the *Diplotaxis* parent: D – OD 77 (BC_1_) hairy buds; E - OD 88 (BC_1_) early drying out and death of the buds; F – pointed, hairy buds; G – OD 97 (BC_2_) fused bud cone, anthers are partially transformed into carpeloid structures; H – OD 121 (BC_2_) abnormal carpel development of three different flowers; I – OD 114 (BC_2_) fused bud cone; J – OD 148 (BC_5_) fused bud cone, large succulent anthers without pollen; K – OD 146 (BC_5_) browed petals in opening buds; L – OD 167 (BC_6_) furrowed mature flower buds;

Supplementary Figure 2 3D reconstruction and 2D cross-section of seed clusters using 3D X-ray CT. *Cluster A:* Normal seed with well-developed embryo, where the embryo completely fills the seed cavity. Embryo volume and specific weight is high, empty space low. *Cluster B:* Pre-germinating seed with only low-density embryo tissue and a high proportion of empty space resulting in a low specific weight. The arrow point to the ruptured seed coat and the emerging embryo. *Cluster C1:* Large seed with an underdeveloped embryo leading to a high percentage of empty space in the seed (“underfilled seeds; arrow). Embryo volume and specific weight are relatively low. This cluster appear to be distinct from Cluster C2, except for embryo volume. *Cluster C2:* Seed with a relatively small volume, which have a “shriveled up” surface as measured by the relative seed surface ratio. They display a relatively small, underdeveloped embryo, tightly covered by pericarp, which is similar in volume to Cluster C1. The specific weight is high due to the seed volume consisting primarily of dense embryo tissue (arrow right). Empty space is very low due to the tight “wrapping” of the small embryo by pericarp (arrow left). *Cluster D:* Small seed having the highest relative seed surface ratio and consisting only of pericarp (arrow left), with no or minimal embryo tissue present (arrow right).

Supplementary Figure 3 Clustering analysis supports the presence of five distinct seed clusters. The similarities between clusters derived from hierarchical clustering and those defined with selected seed level parameters was evaluated using the Adjusted Rand Index (ARI). An ARI of 0.61 indicated overlap between the clustering results, suggesting that external and internal seed characteristics capture largely consistent seed quality patterns. **(A)** Hierarchical clustering showing five clusters. **(B)** Light Gradient Boosting Machine (LightGBM) predicted the defined cluster based on external and internal seed parameters with an accuracy of 98.5%. **(C, D)** Principal Component Analysis (PCA) visualizing hierarchical cluster **(C)** and defined cluster **(D)**. For the defined cluster seeds that could not be clearly assigned to any cluster were classified as border cases.
