# Supplementary material for: Overcoming serious incompatibilities resulting from intergeneric hybridisation between Diplotaxis tenuifolia and Brassica oleracea to develop CMS wall-rocket
Source: Front Plant Sci. 2026 Aug 7;17:1872935. doi: 10.3389/fpls.2026.1872935 (PMC13493536; doi:10.3389/fpls.2026.1872935)
Supplement: Supplementary file 1 [file DataSheet1.pdf]

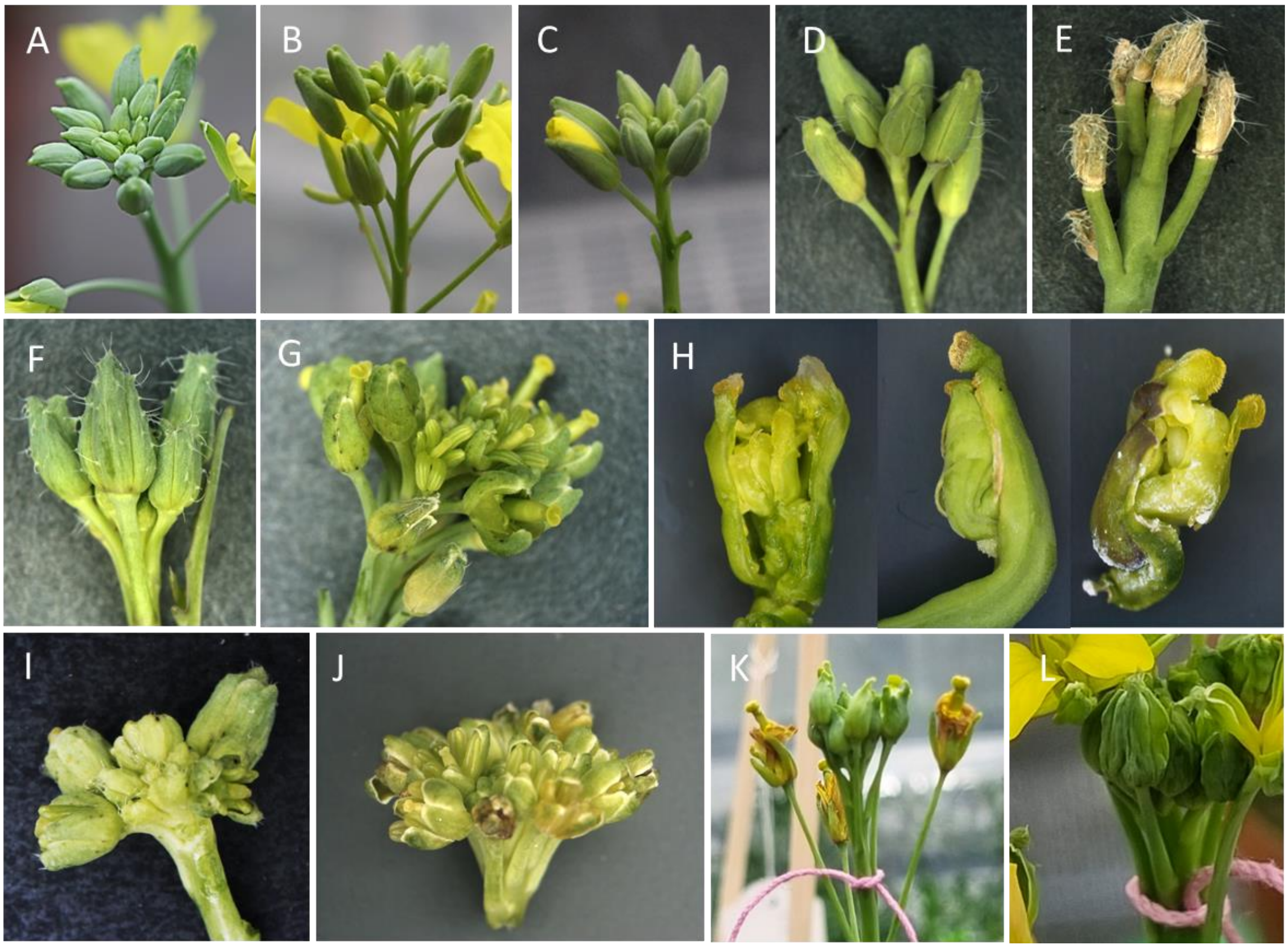

Figure S1 Flower bud architecture of *Diplotaxis tenuifolia* and bud abnormalities in selected progenies of interspecific hybrids between individual *Brassica oleracea* x *Diplotaxis tenuifolia* (OD) lines

A-C normal developed flower buds (control): **A** – *Diplotaxis tenuifolia;* **B** – OD 1 (F_1); **C** – OD 38 (BC_1)

D-L plants with morphological abnormalities compared to the *Diplotaxis* parent: **D** – OD 77 (BC_1) hairy buds; **E** - OD 88 (BC_1) early drying out and death of the buds; **F** – pointed, hairy buds; **G** – OD 97 (BC_2) fused bud cone, anthers are partially transformed into carpeloid structures; **H** – OD 121 (BC_2) abnormal carpel development of three different flowers; **I** – OD 114 (BC_2) fused bud cone; **J** – OD 148 (BC_5) fused bud cone, large succulent anthers without pollen; **K** – OD 146 (BC_5) browed petals in opening buds; **L** – OD 167 (BC_6) furrowed mature flower buds;
