# Supplementary material for: Overcoming serious incompatibilities resulting from intergeneric hybridisation between Diplotaxis tenuifolia and Brassica oleracea to develop CMS wall-rocket
Source: Front Plant Sci. 2026 Aug 7;17:1872935. doi: 10.3389/fpls.2026.1872935 (PMC13493536; doi:10.3389/fpls.2026.1872935)
Supplement: Supplementary file 2 [file DataSheet4.pdf]

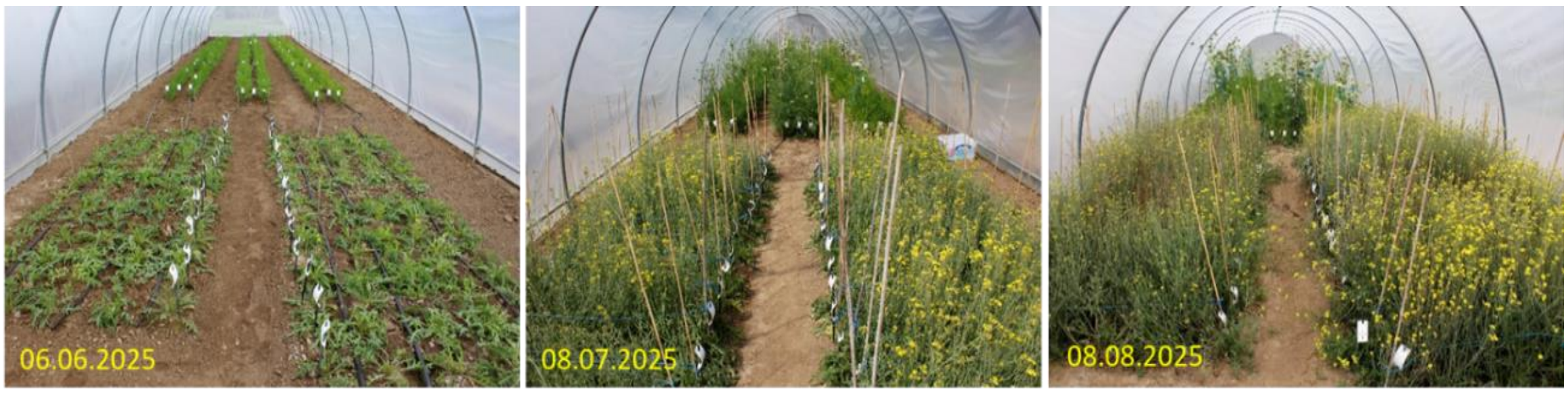

Figure S4: OD 197-202 hybrids (BC_7) growing in a plastic tunnel inclusive *D. tenuifolia* controls (Dt 01, Dt 02, arrows)
