# Supplementary material for: Overcoming serious incompatibilities resulting from intergeneric hybridisation between Diplotaxis tenuifolia and Brassica oleracea to develop CMS wall-rocket
Source: Front Plant Sci. 2026 Aug 7;17:1872935. doi: 10.3389/fpls.2026.1872935 (PMC13493536; doi:10.3389/fpls.2026.1872935)
Supplement: Supplementary file 3 [file DataSheet5.pdf]

Table S1: Seed quantity and quality harvested on BC_5 plants manually backcrossed with *Diplotaxis tenuifolia*

Pedigree	Gen.	ID	Flowers crossed	No of siliques harvested		Seed cluster harvested in total (n) and per silique (Mean ± SD)										
						total		normal (A)		pre-germinated (B)		shrunken (C)		dried (D)		% normal seeds
BC4			n	n	%	n	Mean ± SD	n	Mean ± SD	n	Mean ± SD	n	Mean ± SD	n	Mean ± SD	per silique
OD 142	BC5	OD 143	54	47	87.1	368	7.8 ± 3.1 d	27	0.6 ± 0.4 c	10	0.4 ± 0.9 b	110	2.6 ± 1.1 b	221	4.5 ± 2.3 cd	7,34
OD 142	BC5	OD 144	50	49	98.0	1517	30.9 ± 7.2 b	123	2.6 ± 1.7 b	13	0.3 ± 0.3 b	1075	24.4 ± 8.5 a	306	5.3 ± 4.1 cd	8,11
OD 142	BC5	OD 145	42	21	50.0	1012	48.2 ± 9.5 a	175	8.5 ± 1.5 a	22	1.4 ± 1.4 a	429	19.1 ± 6.5 a	386	18.1 ± 4.9 a	17,29
OD 142	BC5	OD 146	32	32	100.0	1005	31.4 ± 10.1 b	124	3.2 ± 2.7 b	11	0.5 ± 0.5 b	746	22.8 ± 8.5 a	124	3.8 ± 3.1 d	12,34
OD 142	BC5	OD 147	64	62	96.9	1272	20.5 ± 4.8 c	245	3.9 ± 0.7 b	34	0.7 ± 0.3 ab	435	7.8 ± 2.7 b	558	9.1 ± 2.5 bc	19,26
OD 142	BC5	OD 158	33	27	81.8	690	25.5 ± 12.4 c	98	2.9 ± 3.1 b	0	0.0 ± 0.0 c	280	8.1 ± 6.6 b	312	11.7 ± 6.9 b	14,20
			257	238	92.6	5864		792		90		3075		1907		13,51

Different letters show significance at $P < 0.05$
