# Supplementary material for: Overcoming serious incompatibilities resulting from intergeneric hybridisation between Diplotaxis tenuifolia and Brassica oleracea to develop CMS wall-rocket
Source: Front Plant Sci. 2026 Aug 7;17:1872935. doi: 10.3389/fpls.2026.1872935 (PMC13493536; doi:10.3389/fpls.2026.1872935)
Supplement: Supplementary file 4 [file DataSheet6.pdf]

Table S2: Seed quantity and quality harvested on BC_6 plants manually backcrossed with *Diplotaxis tenuifolia*

pedigree	Gen.	ID	Flowers crossed	No of siliques harvested		Seed cluster harvested in total (n) and per silique (Mean ± SD)										
						total		normal (A)		pre-germinated (B)		shrunken (C)		dried D)		% normal seeds per silique
			n	n	%	n	Mean ± SD	n	Mean ± SD	n	Mean ± SD	n	Mean ± SD	n	Mean ± SD	
OD 143	BC6	OD 162	33	0	0,0	0		0		0		0		0		
OD 144	BC6	OD 163	27	15	55,6	485	31.4 ± 12.4 ab	39	2.3 ± 1.7 bcd	5	0.3 ± 0.4 ab	220	15.4 ± 9.2 bc	221	13.3 ± 8.1 a	8.0
OD 146	BC6	OD 164	50	48	96,0	1182	25.1 ± 3.4 b	94	2.5 ± 1.3 bcd	0	0.0 ± 0.0 b	714	15.1 ± 2.6 bc	374	7.5 ± 1.8 bc	7.9
OD 146	BC6	OD 165	23	21	91,3	798	38.8 ± 10.1 a	103	5.2 ± 2.5 ab	8	0.4 ± 0.6 ab	496	24.7 ± 8.7 ab	191	8.5 ± 2.8 ab	12.9
OD 147	BC6	OD 166	79	59	74,7	674	10.6 ± 3.6 c	24	0.4 ± 0.9 d	1	0.1 ± 0.1 b	98	1.9 ± 1.9 d	551	8.2 ± 2.9 ab	3.6
OD 147	BC6	OD 167	64	61	95,3	509	8.2 ± 2.5 c	112	2.1 ± 0.8 cd	28	0.5 ± 0.5 ab	151	2.3 ± 1.6 d	218	3.4 ± 1.3 c	22.0
OD 147	BC6	OD 168	43	37	86,0	855	24.2 ± 5.9 b	159	4.4 ± 1.5 abc	20	0.7 ± 0.6 a	326	9.5 ± 4.7 cd	350	9.6 ± 1.2 ab	18.6
OD 146	BC6	OD 169	60	57	95,0	2396	41.3 ± 11.6 a	356	6.5 ± 2.8 a	7	0.1 ± 0.1 ab	1705	29.1 ± 9.0 a	328	5.5 ± 1.9 bc	14.8
OD 146	BC6	OD 170	50	42	84,0	2047	46.5 ± 13.5 a	297	7.0 ± 2.0 a	1	0.1 ± 0.1 b	1504	32.9 ± 10.9 a	245	6.5 ± 3.4 bc	14.5
			429	340	79,3	8946		1184		70		5214		2478		12.8

Different letters show significance at $P < 0.05$
