# Supplementary material for: Overcoming serious incompatibilities resulting from intergeneric hybridisation between Diplotaxis tenuifolia and Brassica oleracea to develop CMS wall-rocket
Source: Front Plant Sci. 2026 Aug 7;17:1872935. doi: 10.3389/fpls.2026.1872935 (PMC13493536; doi:10.3389/fpls.2026.1872935)
Supplement: Supplementary file 5 [file DataSheet7.pdf]

Table S3: Seed quantity and quality harvested on BC_7 plants manually backcrossed with *Diplotaxis tenuifolia*

pedigree	Gen.	ID	Flowers crossed	No of siliques harvested		Seed cluster harvested in total (n) and per silique (Mean ± SD)										
						total		normal (A)		pre-germinated (B)		shrunken (C)		dried (D)		% normal seeds per silique
			n	n	%	n	Mean ± SD	n	Mean ± SD	n	Mean ± SD	n	Mean ± SD	n	Mean ± SD	
OD 165	BC7	OD 174	33	31	93.9	916	30.0 ± 5.4 a	174	5.3 ± 1.4 ab	173	5.1 ± 3.2 a	348	12.4 ± 7.7 bc	221	7.2 ± 0.4 b	19.0
OD 165	BC7	OD 175	35	28	80.0	775	24.0 ± 12.9 a	82	2.8 ± 0.6 bc	15	0.4 ± 0.5 b	367	11.3 ± 6.6 c	311	9.8 ± 5.8 b	10.6
OD 165	BC7	OD 176	31	27	87.1	840	30.5 ± 2.8 a	146	5.6 ± 2.1 ab	10	0.3 ± 0.3 b	465	17.1 ± 1.7 bc	219	7.5 ± 3.0 b	17.4
OD 165	BC7	OD 177	30	24	80.0	836	35.5 ±6.3 a	155	7.1 ± 2.3 ab	37	1.7 ± 1.6 b	440	18.9 ± 7.8 bc	204	7.8 ± 2.4 b	18.5
OD 169	BC7	OD 178	41	32	78.1	853	23.7 ± 6.7 a	134	3.3 ± 2.3 bc	4	0.2 ± 0.2 b	570	15.6 ± 5.6 bc	145	4.6 ± 2.6 b	15.7
OD 169	BC7	OD 179	57	51	89.5	2314	46.7 ± 11.9 a	318	6.3 ± 2.5 ab	9	0.2 ± 0.2 b	1801	36.1 ± 8.8 ab	186	4.0 ± 1.8 b	13.7
OD 169	BC7	OD 180	46	37	80.4	1646	44.6 ± 6.5 a	325	8.4 ± 2.1 a	7	0.2 ± 0.1 b	1130	30.8 ± 5.2 ab	184	5.2 ± 0.7 b	19.7
OD 169	BC7	OD 181	45	40	88.9	1651	41.5 ± 7.4 a	191	4.7 ± 2.9 ab	5	0.1 ± 0.3 b	1223	30.8 ± 6.8 ab	232	5.9 ± 1.4 b	11.6
OD 170	BC7	OD 182	55	48	87.3	2310	48.0 ± 2.5 a	113	2.3 ± 0.7 c	0	0	1064	20.6 ± 10.7 bc	1133	25.2 ± 9.3 a	4.9
OD 170	BC7	OD 183	46	42	91.3	2015	47.3 ± 2.5 a	287	5.9 ± 2.5 ab	0	0	1480	34.0 ± 3.3 ab	248	7.3 ± 3.6 b	14.2
OD 170	BC7	OD 184	45	37	82.2	1843	38.2 ± 25.0 a	94	2.1 ± 1.3 c	0	0	1331	26.3 ± 18.7 bc	418	9.8 ± 10.7 b	5.1
OD 170	BC7	OD 185	55	53	96.4	2642	46.9 ± 6,4 a	202	3.4 ± 1.7 bc	0	0	2189	38.7 ± 5.7 a	251	4.8 ± 1.8 b	7.6
			559	490	87.6	18641		3509		260		12408		3782		18.8

Different letters show significance at $P < 0.05$
