# Supplementary material for: Overcoming serious incompatibilities resulting from intergeneric hybridisation between Diplotaxis tenuifolia and Brassica oleracea to develop CMS wall-rocket
Source: Front Plant Sci. 2026 Aug 7;17:1872935. doi: 10.3389/fpls.2026.1872935 (PMC13493536; doi:10.3389/fpls.2026.1872935)
Supplement: Supplementary file 6 [file Image2.pdf]

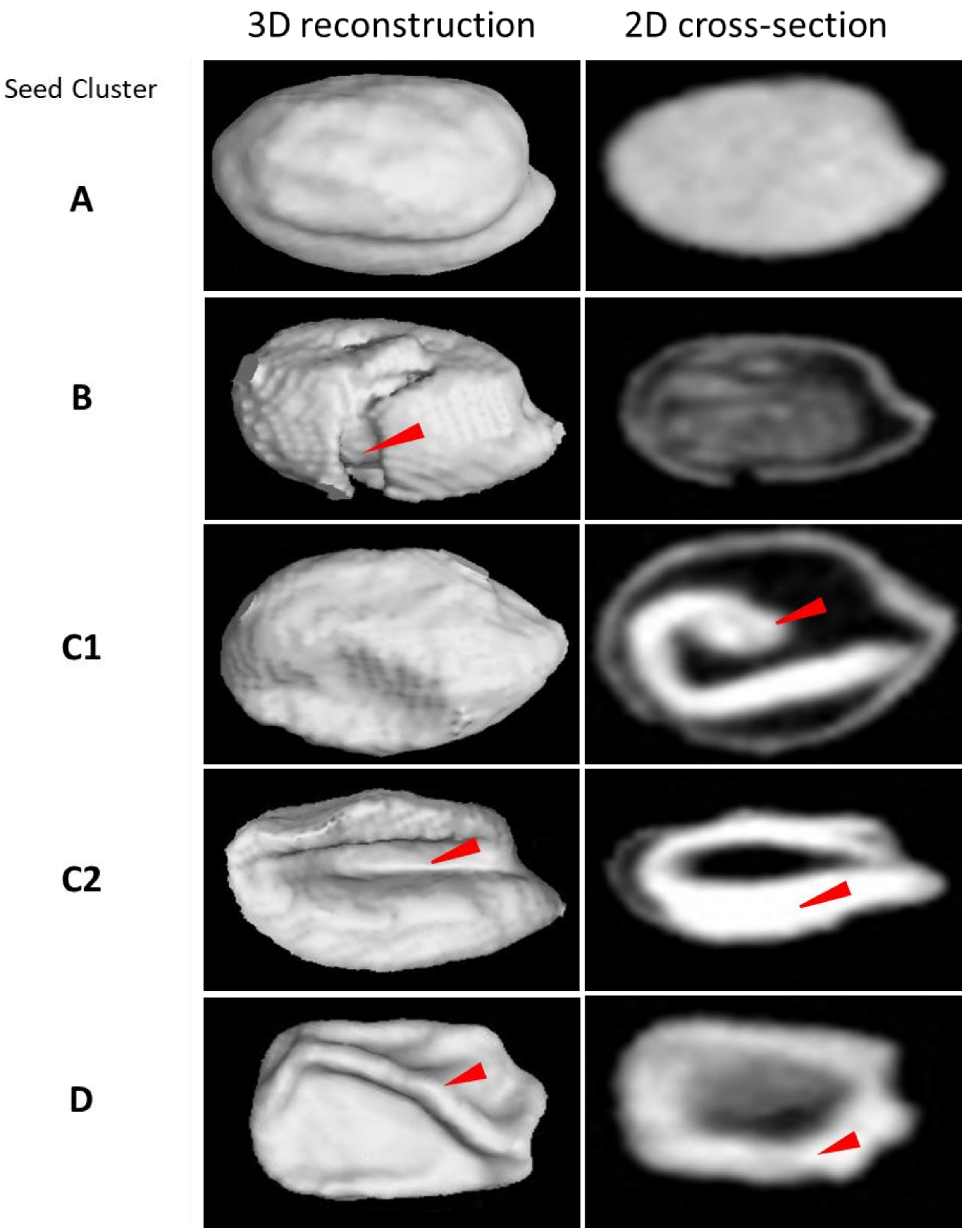

Figure S2 3D reconstruction and 2D cross-section of seed clusters using 3D Xray CT

Cluster A: Normal seed with well-developed embryo, where the embryo completely fills the seed cavity. Embryo volume and specific weight is high, empty space low.

Cluster B: Pre-germinating seed with only low-density embryo tissue and a high proportion of empty space resulting in a low specific weight. The arrow point to the ruptured seed coat and the emerging embryo.

Cluster C1: Large seed with an underdeveloped embryo leading to a high percentage of empty space in the seed ("underfilled seeds")(arrow). Embryo volume and specific weight are relatively low. This cluster appear to be distinct from Cluster C2, except for embryo volume.

Cluster C2: Seed with a relatively small volume, which have a "shriveled up" surface as measured by the relative seed surface ratio. They display a relatively small, underdeveloped embryo, tightly covered by pericarp, which is similar in volume to Cluster C1. The specific weight is high due to the seed volume consisting primarily of dense embryo tissue (arrow right). Empty space is very low due to the tight "wrapping" of the small embryo by pericarp (arrow left).

Cluster D: Small seed having the highest relative seed surface ratio and consisting only of pericarp (arrow left), with no or minimal embryo tissue present (arrow right).
