# Supplementary material for: Overcoming serious incompatibilities resulting from intergeneric hybridisation between Diplotaxis tenuifolia and Brassica oleracea to develop CMS wall-rocket
Source: Front Plant Sci. 2026 Aug 7;17:1872935. doi: 10.3389/fpls.2026.1872935 (PMC13493536; doi:10.3389/fpls.2026.1872935)
Supplement: Supplementary file 7 [file Image3.pdf]

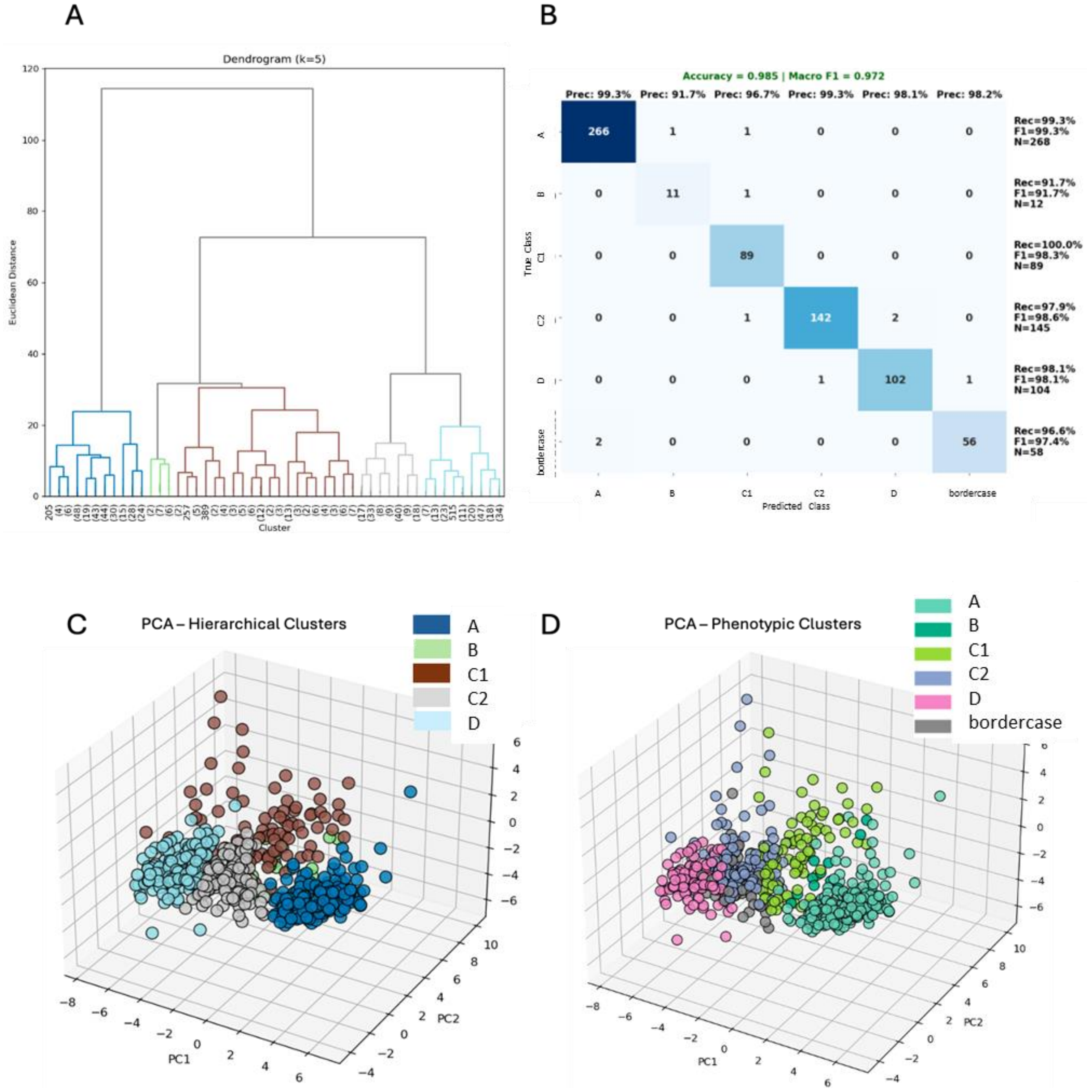

Figure S3: Clustering analysis supports the presence of five distinct seed clusters. The similarities between clusters derived from hierarchical clustering and those defined with selected seed level parameters was evaluated using the Adjusted Rand Index (ARI). An ARI of 0.61 indicated overlap between the clustering results, suggesting that external and internal seed characteristics capture largely consistent seed quality patterns. (A) Hierarchical clustering showing five clusters. (B) Light Gradient Boosting Machine (LightGBM) predicted the defined cluster based on external and internal seed parameters with an accuracy of 98.5%. (C, D) Principal Component Analysis (PCA) visualizing hierarchical cluster (C) and defined cluster (D). For the defined cluster seeds that could not be clearly assigned to any cluster were classified as border cases.
